# *dlmoR*: An Open-Source R Package for the Dim-Light Melatonin Onset (DLMO) Hockey-Stick Method

**DOI:** 10.1177/07487304251389994

**Published:** 2026-02-12

**Authors:** Salma M. Thalji, Manuel Spitschan

**Affiliations:** * Translational Sensory and Circadian Neuroscience, Max Planck Institute for Biological Cybernetics, Tübingen, Germany; † Chronobiology & Health, TUM School of Medicine and Health, Technical University of Munich, Munich, Germany; ‡ TUM Institute for Advanced Study (TUM-IAS), Technical University of Munich, Garching, Germany

**Keywords:** dim-light melatonin onset (DLMO), circadian rhythm, biological clocks, sleep, open-source software, algorithms

## Abstract

The dim-light melatonin onset (DLMO) is a commonly used circadian marker indicating the start time of evening melatonin synthesis in humans. Several quantitative techniques have been developed to determine DLMO from melatonin time series, including fixed- or variable-threshold techniques and the hockey-stick method developed by Danilenko et al (2014). Here, we introduce *dlmoR*, an open-source (MIT License) implementation of the hockey-stick method written in R. Our clean-room implementation follows the original algorithm description, supported by iterative validation against the existing binary executable. We benchmarked *dlmoR* on 112 melatonin time series data sets from two independent studies and found high agreement with the reference implementation: mean discrepancies were −1.482±21.7 min for the Heinrichs and Spitschan (2025) data set and 1.165±28.5 min for the Blume et al. (2024) data set, with circular correlation coefficients of 0.964 and 0.986, respectively. Paired *t*-tests ( p>0.05 ) indicated no systematic difference or bias between methods. Beyond reproducing the hockey-stick algorithm, *dlmoR* adds capabilities absent from the original executable, including interactive visual diagnostics and bootstrapped confidence intervals, offering qualitative and quantitative views of estimation uncertainty. It supports programmatic, reproducible analysis of melatonin profiles, including batch processing and parameter manipulation. Leveraging this flexibility, we evaluated the sensitivity of the hockey-stick algorithm to controlled changes in sampling schedules, threshold levels, data completeness, and noise. Moderate changes, such as small timing jitter, limited data loss, or modest threshold shifts, kept estimates stable within ±10 min, whereas pronounced alterations to sampling schedules, large multi-point deletions, or substantial threshold changes delayed estimates by over 40 min or prevented estimation. This analysis reveals fundamental limitations in the algorithm’s internal mechanics, particularly in how it identifies the onset window and models the melatonin rise, and underscores the need for new uncertainty-aware approaches to DLMO estimation.

## Graphical abstract

**Graphical abstract:**
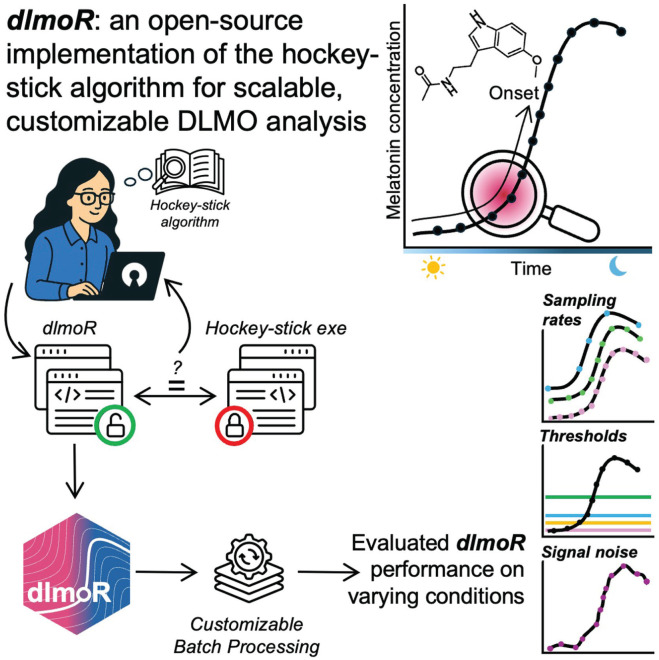
*dlmoR* is an open-source R implementation of the hockey-stick algorithm for dim light melatonin onset (DLMO)estimation. In this paper, we introduce *dlmoR*, validate its agreement with the reference implementation, and evaluate the hockey-stick algorithm’s sensitivity to data sampling rate, threshold values, data loss, and signal noise.

The dim-light melatonin onset (DLMO) is the most reliable and widely used marker of circadian phase. By marking the onset of melatonin synthesis under dim-light conditions in time, the DLMO provides precise insights into the synchronization of an individual’s circadian system with the 24-h light/dark cycle (Pandi-Perumal et al., 2007; Keijzer et al., 2014). The DLMO is determined through bioanalytic assays of melatonin levels in saliva, plasma, or urine (Benloucif et al., 2008; Pandi-Perumal et al. 2007). The DLMO is instrumental in assessing phase delays, advances, and other deviations from expected circadian timing and plays a key role in research and clinical practice, aiding in the diagnosis and treatment of circadian rhythm sleep disorders, mood disorders, and seasonal affective disorder (Pandi-Perumal et al., 2007; Keijzer et al., 2014).

Various methods have been developed to calculate DLMO, including fixed thresholds, dynamic thresholds, and visual estimation. However, these approaches have limitations: fixed thresholds may fail in low melatonin secretors, dynamic thresholds struggle with missing or inconsistent baseline data, and visual estimation is subjective and prone to variability (Kennaway, 2023; Glacet et al., 2023). The hockey-stick algorithm (Danilenko et al., 2014) addresses these challenges by modeling melatonin rise as a piecewise linear-parabolic curve. This approach provides a more objective and reliable estimate of DLMO and has demonstrated superior accuracy, repeatability, and robustness compared to traditional methods (Danilenko et al., 2014; Glacet et al., 2023).

A Windows software implementation was developed and released by the authors (Danilenko and Verevkin, 2020). However, its usability is limited by the constraints of being a binary executable. This closed-source format does not allow researchers to inspect, modify, or directly interact with the underlying implementation of the algorithm. In addition, executable files without an API do not integrate seamlessly with widely used analytical workflows.

This paper introduces *dlmoR*, an open-source R package implementing the hockey-stick method. The package provides transparency into the algorithm, integrates seamlessly into analytical pipelines, and allows parameter adaptation for diverse experimental needs. Its open-source license fosters community contributions and ongoing improvements (Thalji and Spitschan, 2026).

## Methods

### Algorithm Analysis and Prior Implementation

Our open-source R implementation of the hockey-stick algorithm is based on our study of the original description by Danilenko et al. (2014), complemented by insights from personal communication with the author. A Windows executable of the algorithm, *hockeystickexe* (Danilenko and Verevkin, 2020), is available as a free download but only in closed-source form. We adopted this executable as the reference against which our implementation was validated.

### Iterative Development

Guided by the journal article and author correspondence, we created an initial R implementation (*dlmoR*) and iteratively compared its outputs with *hockeystickexe* on identical data sets. Each discrepancy highlighted aspects of the algorithm requiring adjustment. Through successive refinements, our implementation converged toward the behavior and objectives of the original algorithm.

### Implementation Details

**Figure 1.**
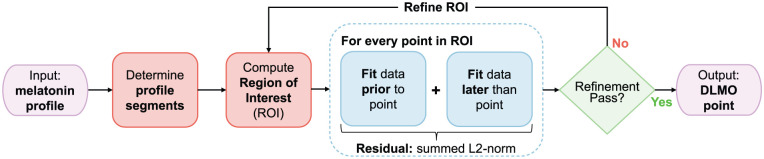
Flowchart of the *dlmoR* algorithm, which determines the dim light melatonin onset (DLMO) timestamp by identifying the inflection point in a melatonin profile. The algorithm first segments raw melatonin concentration data and defines a region of interest (ROI) for detecting melatonin rise. Candidate breakpoints within the ROI are evaluated in two phases: an initial coarse-grid pass across all points, followed by a fine-grid refinement phase restricted to the 10% with the lowest residuals from the initial pass. For each candidate, data before and after the point are fit separately, and residual error is computed as the summed L2-norm (Euclidean distance) between observed and fitted values. The point with the minimum residual from the refinement phase is identified as the DLMO point.

We chose to implement the hockey-stick algorithm in R (R Core Team, 2024) due to its wide use, flexibility, open-source availability cross-platform support and modularity. Our implementation was designed in a modular fashion, with each module corresponding to a distinct component of the framework (see Figure 1).

Each module was implemented as a separate script or set of scripts, focusing on the following key functionalities:

- *Preprocessing*: Functions handle the input data formatting and any required preprocessing steps, ensuring compatibility with the algorithm.
- *Core algorithm*: The core computation logic of the algorithm is encapsulated in dedicated modules, ensuring clarity and focus on the primary processing tasks.
- *Postprocessing and visualization*: Separate modules generate outputs, including tables and visualizations to aid in interpreting the results.

The implementation utilizes several R packages to streamline development and enhance functionality, including *dplyr* for data manipulation, *tidyverse* for efficient data handling, and *ggplot2* for visualization (Wickham et al., 2023, 2019; Wickham, 2016). Custom functions were developed where necessary to meet the specific requirements of the algorithm.

A comprehensive documentation system was embedded within the code, including inline comments and a user guide, to promote reproducibility and ease of use. In addition, basic unit tests and validation checks were integrated into the workflow to ensure reliability and correctness. Error handling mechanisms, such as logging and exception handling, are also included to provide robust operational stability.

### Performance Evaluation

To evaluate the performance of the *dlmoR* implementation, its concordance in DLMO estimation was assessed against the hockey-stick executable (*hockeystickexe*).

#### Data sets and Sampling Protocols

Two data sets comprising a total of 120 melatonin concentration time profiles were analyzed to determine DLMO using both methods.

- Blume et al. (2024) *data set*: A total of 96 melatonin concentration time profiles, each consisting of 14 data points, were collected from 16 participants, each of whom attended three separate laboratory visits. Saliva samples (≥1 mL) were collected using Salivettes (Sarstedt, Nürnberg, Germany) at 30-min intervals.
- Heinrichs and Spitschan (2025) *data set*: A total of 24 melatonin concentration time profiles, each consisting of 23 data points, were collected from 12 participants during 2-day laboratory visits. Each participant contributed two profiles, with saliva samples (≥1 mL) collected at 45-min intervals.

The exact protocols and research questions asked in these two studies are irrelevant to the purpose of determining the DLMO and are therefore not further described. In both data sets, melatonin concentrations were quantified using a radioimmunoassay (RIA, RK-DSM2, NovoLytiX GmbH, Switzerland) with a limit of quantification of 0.5–50 pg/mL, a detection limit of 0.2 pg/mL, a mean intra-assay precision of 7.9%, and a mean inter-assay precision of 9.8%.

#### Thresholds for DLMO Analysis

For the performance evaluation, we assumed the following ascending level thresholds. For the Blume et al. (2024) data set, an ascending-level threshold of 5 pg/mL, consistent with the methodology outlined in Blume et al. (2024), was applied. For the Heinrichs and Spitschan (2025) data set, the default ascending level threshold of 2.3 pg/mL was used.

#### Data Exclusion

Eight of the 96 profiles in the Blume et al. (2024) data set did not produce DLMO estimates using either method for one of three reasons: the profile was ambiguous, with no distinct baseline or discernible melatonin rise; the melatonin concentrations of the entire profile remained above the pre-defined threshold; or the entire profile remained below the pre-defined threshold. These data were excluded, leading to the inclusion of 88 time series from the Blume et al. (2024) data set and 24 time series from the Heinrichs and Spitschan (2025) data set, yielding a total of 112 data sets included in the analysis.

#### Analysis Workflow

The analysis workflow differed between *dlmoR* and *hockeystickexe*. In *dlmoR*, melatonin profiles from both data sets were batch-processed using a custom script. The script parsed all melatonin profiles and determined the DLMO timestamp for each profile based on the user-defined ascending level threshold. In addition, *dlmoR* saved a data structure for each profile containing the calculated DLMO timestamp, profile fit parameters, and the resulting plot. For analysis with *hockeystickexe*, each of the 112 melatonin profiles was manually entered into the *hockeystickexe* graphical user interface (GUI). The analysis for each profile was initiated by a button click and the resulting plot was saved manually. The DLMO timestamp printed in the GUI was manually tabulated in a spreadsheet alongside the corresponding sample name.

### Code, Materials, and Data Availability

All code, materials, and data are available on our GitHub repository (https://github.com/tscnlab/ThaljiEtAl_JBiolRhythms_2026) and archived on Zenodo (DOI: 10.5281/zenodo.18390504). dlmoR is available on GitHub (https://github.com/tscnlab/dlmoR) under the MIT License, archived on Zenodo (DOI: 10.5281/zenodo.18390029); RRID:SCR_027906.

## Description of the Hockey-Stick Algorithm

### Overall Overview

The hockey-stick algorithm comprises three distinct phases (see Figure 1). First, the input melatonin profile is validated and segmented into baseline and ascending intervals. Next, a region of interest (ROI) is established, within which the DLMO point will be sought in the subsequent phase. Finally, a piecewise function is fitted to the segmented melatonin profile, and the DLMO point is identified as the location within the ROI where the fitted profile yields the lowest residual. This time point is then reported as the output.

In the description that follows, we detail our interpretation of the algorithm, grounded in the original formulation by Danilenko et al. (2014), prior personal communication with the authors, and insights gained during the iterative development of our implementation. In this description, *node* refers to a melatonin data point, and *segment* refers to the region between a left node and a right node, including the right node but excluding the left node. More precise implementation details are provided as pseudocode in Supplementary section A (online).

The hockey-stick algorithm relies on several pre-defined parameter values—some documented by Danilenko et al. (2014), others not explicitly described. We reconstructed the rationale for these parameters through a close reading of the original description, informed by our correspondence with Prof. Danilenko and by systematic empirical testing during the development of *dlmoR*. This rationale is summarized in Table S1 of Supplementary section B (online). The open-source nature of *dlmoR* further allows advanced users to inspect and modify these parameters, enabling direct exploration of how such changes influence the DLMO estimation process.

### Validating the Melatonin Profile

First, the input melatonin profile is validated to ensure that it meets the necessary criteria for DLMO analysis using the hockey-stick method. Profiles are included in the analysis only if they exhibit a rise above a defined threshold (default value: 2.3 pg/mL, which is modifiable). If this criterion is not met, a warning is triggered, indicating the absence of a dynamic portion in the profile (“no dynamic part”). In addition, profiles with only one or two data points are considered insufficient for analysis and a corresponding warning is issued (“insufficient number of data points”). Finally, in cases where the profile shows a rise, but terminates with data points below the threshold, these terminal points are excluded from the analysis.

### Determining the Profile Segments

#### Defining the Base Region

Any segment below the threshold with a zero or negative slope ( tanθ≤0 ), or crossing the threshold in a downward direction, is classified as part of the *base* section. The *base* section also includes all segments located to the left of this segment. If a segment descends across the threshold or if the difference between the values of the two nearest nodes in the *base* section exceeds half the threshold value, the algorithm flags an inconsistency. In such cases, the following output is generated at the end of the module (“Profile consistency at base part: no”).

#### Defining the Ascending Region

The ascending region is defined as follows:

1. The segment that crosses the ascending threshold, along with all subsequent segments above the threshold, is classified as the *ascending* part. If the profile exhibits two rises above the threshold with an interval of less than 2 h (default value), only the segments from the second rise onward are considered as part of the *ascending* section. The interval length is customizable and can be adjusted by the user.
2. In the portion of the profile not labeled as *base*, the *ascending* part also includes the steepest segment, all segments with a steepness exceeding half that of the steepest segment, and any segments situated between these steep segments. This rule does not exclude any segments identified in rule (1) but may add additional segments, including those below the threshold, if they meet the steepness criteria.
3. If the segment immediately preceding the first *ascending* segment (as determined by rules 1 and 2) has a steepness of at least half that of the *ascending* segment, it is also classified as part of the *ascending* section. Rule (3) is then reapplied iteratively to include additional segments if the same condition holds.

#### Truncating the Ascending Region

Three distinct rules are applied to truncate the *ascending* region, ensuring that the melatonin rise considered for determining the DLMO is both rapid and consistent.

**Figure 2.**
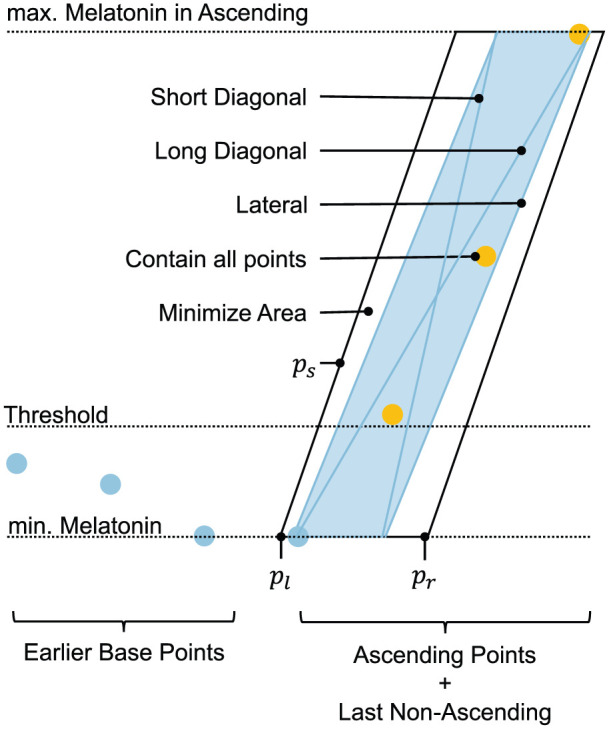
An illustration of the properties of a parallelogram used in the *parallelogram rule* for truncating the *ascending* region. The blue points represent *baseline* data, while the yellow points represent *ascending* data. The construction and optimization process involves determining the lower-left x-coordinate (*pl*), the lower-right x-coordinate (*pr*), and the slope of the lateral edge (*ps*). Annotations indicate the properties of an optimal parallelogram (i.e., the one with the smallest area) fitted around all ascending points and the last data point preceding the *ascending* segment.

1. First, the rightmost segment of this region is excluded from the *ascending* portion if it has a zero or negative slope ( tanθ≤0 ).
2. The parallelogram rule: After applying rule (1), segments of the *ascending* melatonin profile that rise too slowly are truncated using the properties of an optimal parallelogram fitted around all *ascending* points and the last data point preceding the *ascending* region (see Figure 2). Specifically, the ratio of the slope of the parallelogram’s longest diagonal to the slope of its lateral edge must be greater than 1/2. If this condition is not met, the last segment of the *ascending* profile is truncated. This process is repeated iteratively, with the parallelogram being recalculated after each truncation, until the rule is satisfied.

The construction of this parallelogram is subject to three constraints: (1) the lower edge must be horizontal and pass through the point with the lowest melatonin level, (2) the upper edge must be horizontal and pass through the point with the highest melatonin level, and (3) all *ascending* points must lie within the parallelogram. The term *optimal* refers to a parallelogram with the smallest possible area that satisfies these constraints.

This construction and optimization process involves determining the lower-left x-coordinate of the parallelogram ( $p_{l}$ ), the lower-right x-coordinate ( $p_{r}$ ), and the slope of the lateral edge ( $p_{s}$ ) (see Figure 2). By treating these as free parameters, the first and second constraints are inherently satisfied. The third constraint, that all *ascending* points are encompassed by the parallelogram, is treated as a soft constraint. Mathematically, the optimization can be expressed as follows:

(1) $$\;\arg\;\underset{p}{\max}A\left(p\right)-\lambda C(p,x)$$

where $p=\left(p_{l},p_{r},p_{s}\right)$ is the optimization parameters, x are all data points labeled as *ascending* and the last point prior to the first *ascending* point, A(p) and C(p,x) are the functions that compute the area of the parallelogram and soft-constrained, respectively, and λ>>1 is a weighting scalar. We solve this optimization problem using the SANN algorithm available in the *Optim* package (Pan, 2022) in R. For brevity, the formulation of A(p) , C(p,x) , and the initialization procedure are detailed in Supplementary section A (online), Algorithm 4.

3. The rightmost segment cannot be less than half as steep as the steepest of the remaining *ascending* segments (as defined after applying truncation Rules 1 and 2).

Rules (2) and (3) can be disabled if desired.

#### Adding a Segment to the Left of the Ascending Region

If at this stage all segments belong to the *ascending* region, and the leftmost node is the only one that lies below half the threshold, an additional node is added 30 min to the left of it with a melatonin concentration level equal to either two times lower than the level of the leftmost node or zero, whichever is higher. The added segment is classified as *intermediate* (see below).

#### Truncating the Base Region

If the *base* part begins with nodes lying above the threshold, these nodes are excluded from the *base* part (see Figure 4, Panel A). Although they remain visible in the figure, they are not included in the analysis.

#### Defining the Intermediate Region

Any segments lying between the *base* and *ascending* regions, that belong to neither of the two, are classified as *intermediate*.

### Computing the Bounds of the ROI

The ROI refers to the portion of the melatonin profile that will be parsed in the subsequent stage of the algorithm to identify the DLMO point. First, a line of interest is defined for the left and right bounds of the ROI, and then the upper and lower bounds are defined.

#### Line of Interest

**Figure 3.**
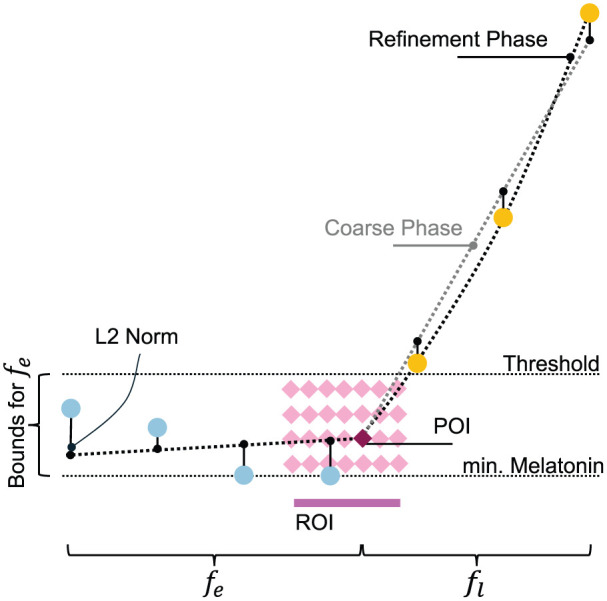
Schematic of the iterative grid search for the DLMO point. The *coarse phase* iterates over the region of interest (ROI), fitting piecewise functions to the *base* ( $f_{e}$ ) and *ascending* ( $f_{l}$ ) profile regions and calculating a composite residual for each point of interest (POI). The *refinement phase* narrows the ROI with finer resolution, and the POI with the lowest residual is identified as the DLMO time point.

To determine the line of interest, there are two scenarios:

1. *No intermediate point present*: If the *base* and *ascending* parts are defined, and no *intermediate* segments are present, the line of interest includes the last half (50%) of the final *base* segment and the first 95% of the first *ascending* segment. This interval is projected onto the time-axis and is denoted by a thick line parallel to the x-axis on the output plot (see Figure 3). If there is only a single *base* node, the line of interest becomes narrower, spanning from this node to the first 95% of the first *ascending* segment.
2. *Intermediate points present*: If *intermediate* segments are present, the line of interest spans from immediately after the last *base* node, includes all *intermediate* segments, and extends to the first 95% of the first *ascending* segment.

#### ROI Definition

The ROI is defined by the bounds of the line of interest, which set its left and right limits, while the upper and lower boundaries are determined by the threshold value and the melatonin level of the lowest node in the profile, respectively (see Figure 3).

### Find the DLMO Point

At this stage, all data points have been categorized into two (or three) groups: *base*, *ascending*, and (when present) *intermediate*. In addition, an ROI has been defined within which we aim to identify the inflection point. Now, our method employs two phases to detect the inflection point: a coarse phase and a refinement phase (see Figure 3).

#### Coarse Phase of DLMO Search

In the coarse phase, we perform a grid search over the ROI. The search uses step sizes of 0.1 in the time dimension (x-values) and 0.2 in the y-values. For each POI in the grid, the following steps are carried out:

1. *Linear fit to earlier points*: A linear model is fit that minimizes the $L_{2}$ -norm for all points occurring earlier than the POI, including the POI itself. The linear fit is constrained to pass through the POI, and its slope is limited to the range of −0.2 to 0.2. In addition, the fitted line cannot exceed the highest y-value (threshold) or fall below the lowest y-value in the fitting domain.
2. *Linear fit to later points*: Similarly, a linear model is fit that minimizes the $L_{2}$ -norm for all points occurring later than the POI, including the POI itself. This line is also constrained to pass through the POI.

The overall cost associated with each POI is calculated as the sum of the residual $L_{2}$ -norms from the two linear fits.^2^

#### Refinement Phase of DLMO Search

**Figure 4.**
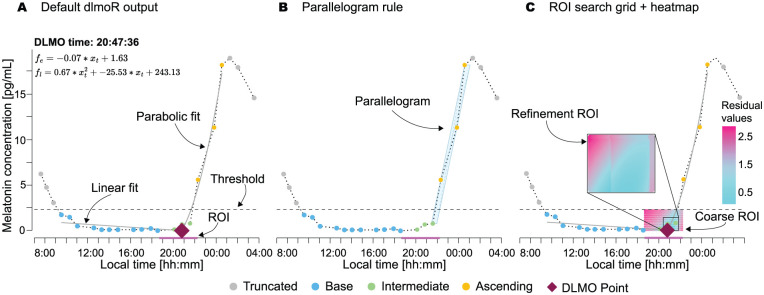
Visualizations of the hockey-stick method implemented in *dlmoR* with an example profile from the Heinrichs and Spitschan (2025) data set. a Default *dlmoR* output, indicating various components of the fitted profile, including annotations of the linear ( $f_{e}$ ) and parabolic ( $f_{l}$ ) fit parameters. b Output including the visualization of the optimal parallelogram used for truncation of the ascending region of the profile. c Output with an overlay of the ROI fit residual heatmap used for DLMO point search.

In the refinement phase, we narrow the ROI to focus on the extrema of the 10% of points with the lowest costs from the coarse phase. This phase is similar in approach but uses finer step sizes of 0.01 in both dimensions to achieve greater precision (see Figure 4).

During the refinement phase, a parabola is fit to the later points. This parabola must satisfy the following constraints: (1) the parabola is required to pass through the POI, (2) the derivative of the parabola must never be zero, (3) the slope of the parabola at the POI must be at least half as steep as the slope of the linear fit identified during the coarse phase, and (4) steeper than the slope of the linear fit for the earlier points.

Generally, for both the coarse and refinement phases, the cost of a point can be expressed as:

(2) $$\;R=f_{e}\left(p_{t},\{x_{t}:x_{t}\leq p_{t}\}\right)+f_{l}\left(p_{t},\{x_{t}:x_{t}\geq p_{t}\}\right)$$

where $x_{t}$ represents all data points and $p_{t}$ is the POI. Specifically:

-
$\left\{x_{t}:x_{t}\leq p_{t}\right\}$ denotes all points earlier than and/or equal to the POI.
-
$\left\{x_{t}:x_{t}\geq p_{t}\right\}$ denotes all points later than and/or equal to the POI.

Here, $f_{e}$ is the linear fit function applied to the earlier data points, and $f_{l}$ is the fit function for the later data points, which can be either linear or parabolic depending on the search phase. Details of this implementation can be found in Supplementary section A (online), Algorithm 9.

This two-phase design, coarse search followed by refinement, follows the original hockey-stick algorithm described by Danilenko et al. (2014), which uses the coarse phase to efficiently locate a promising ROI before applying a finer grid to that reduced region. While the refinement step is more computationally demanding, it is still substantially faster than applying a fine grid across the entire parameter space. In *dlmoR*, both coarse and refined results are retained and reported, whereas *hockeystickexe* outputs only the final refined estimate. This modularity allows *dlmoR* users to run only the coarse phase if a faster, approximate estimate is sufficient (typically under 1 min per profile), or to include the refinement phase for maximum precision (typically 2–3 min per profile), with exact runtimes depending on ROI size and hardware performance.

## Results

### Independent Implementation of the Hockey-Stick Algorithm

Despite the original hockey-stick software being only available as a closed-source Windows executable, we were able to derive an R-based implementation of the hockey-stick algorithm developed by Danilenko et al. (2014).

### Performance Evaluation

We assessed the concordance in DLMO time estimates derived from the two methods—*dlmoR* and *hockeystickexe*—applied to two data sets comprising a total of 120 melatonin time series Blume et al. (2024); Heinrichs and Spitschan (2025), with a total of 112 usable data sets. Circular statistics were employed to evaluate concordance due to the periodic nature of time data.

#### Descriptive Statistics

The circular difference in DLMO time estimates between the two methods were summarized using descriptive statistics, namely the mean and standard deviation, to describe the central tendency and dispersion. The mean difference in DLMO estimates between *dlmoR* and *hockeystickexe* is 1.165±28.5 min for the Blume et al. (2024) data set, and −1.482. ±21.7 min for the Heinrichs and Spitschan (2025) data set.

#### Outliers

Outliers were identified by manually inspecting data profiles with DLMO estimate discrepancies between methods exceeding ±1 h. Of the 112 profiles analyzed, two significant outliers were observed: a DLMO estimate difference of approximately +80 min and one of approximately +240 min, indicating that the *dlmoR* method estimated DLMO timestamps more than 1 h later than *hockeystickexe*. Close inspection of each scenario highlighted two key scenarios, illustrated in Supplementary section C (online) in which *dlmoR* and *hockeystickexe* analysis differs: (1) the handling of spurious above-threshold melatonin rises occurring early in the time profiles, and (2) differing interpretations of multiple rises above threshold between methods. In the case of the melatonin profile with a between-method DLMO difference of 80 min, a single melatonin sample briefly rose above the threshold, followed by a decline and a subsequent steady rise within 1 h (Case 1, see Supplementary section C (online), Figure S1, Panel A). The *hockeystickexe* algorithm fit the earlier single-point rise, whereas *dlmoR* fit the later steady consecutive rise. For the melatonin profile with a +240-min difference, a spurious single-point rise near the beginning of the profile (second point in a 14-point time series) was fit as the DLMO by *hockeystickexe. dlmoR* disregarded this as noise and fit the steady rise occurring 4 h later (Case 2, see Supplementary section C (online), Figure S1, Panel B). Without outliers, the mean discrepancy in DLMO estimate between methods was −2.082 ±11.1 min for the Blume et al. (2024) data set. The small mean differences in both data sets suggest minimal overall difference in DLMO estimate between the methods. The larger standard deviation in the Blume et al. (2024) data set reflects greater variability in the differences compared to the Heinrichs and Spitschan (2025) data set.

#### Method Agreement Within Specific Time Thresholds

**Figure 5.**
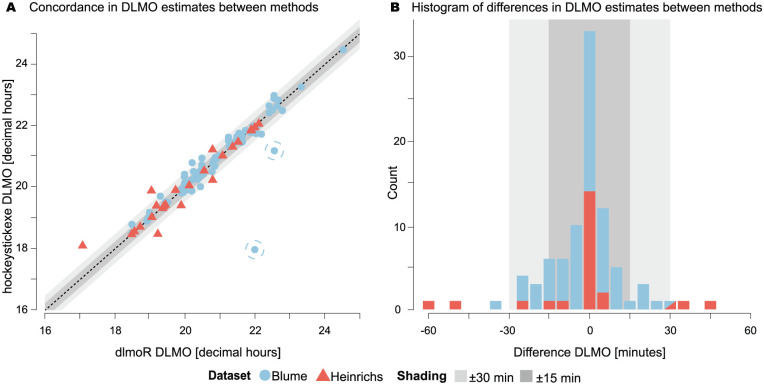
Performance evaluation of *dlmoR* against *hockeystickexe*. a Concordance in DLMO estimate between implementations, with two outliers highlighted by circles (see subsection *Outliers* for details and Supplementary section C (online) for visualization). b Histogram of differences in DLMO estimates between methods.

The agreement between methods was further assessed by categorizing DLMO estimates based on time difference thresholds. For the Blume data set, 46 estimates (52.3 %) were within ±5 min of each other, 69 estimates (78.4%) were within ± 15 min, and the difference in three estimates (3.41%) exceeded ±30 min. For the Heinrichs data set, 16 estimates (66.7%) were within ±5 min, 18 estimates (75%) were within ±15 min, and the difference in 4 estimates (16.7%) exceeded ±30 min (see Figure 5, Panel B) .

#### Concordance and Bias Detection

Circular correlation coefficients and paired t-tests were used to evaluate the strength and significance of agreement and to identify systematic biases between the two methods. Circular correlation values were 0.986 (0.911 with outliers) for the Blume et al. (2024) data set and 0.964 for the Heinrichs and Spitschan (2025) data set, indicating strong agreement between the methods in both data sets (see Figure 5, Panel A). Circular paired *t*-tests yielded *p* values of 0.088 (0.601 with outliers) for the Blume data set and 0.742 for the Heinrichs data set, suggesting no systematic difference or bias between the methods.

*Skewness and bias Analysis* of skewness revealed minor biases in DLMO estimation. The Blume et al. (2024) data set had a skewness of −0.22 (6.37 with outliers), while the Heinrichs and Spitschan (2025) data set had a skewness of −0.796. These values suggest that *dlmoR* tends to provide slightly earlier estimates compared to *hockeystickexe*.

#### Visual Inspection and Residual Analysis

Visual inspection of fitted melatonin profiles revealed two primary contributors of discrepancies between methods, aside from identified outliers:

1. *Parallelogram-based ascending segment truncation*: The methods differed in the truncation of *ascending* segments, leading to the fitting of different sets of points. This impacted the distribution of residual heatmaps and the selection of the DLMO point, which corresponds to the timepoint with the lowest fit residual.
2. *Diffuse ROI heatmap*: The *dlmoR* visualization indicated diffuse ROI heatmaps with multiple points offering similarly good fits (differences on the order of 1 ×10–^2^). Minor variations in fit procedures likely contributed to residual variation. When small deviations in the fits lead to minor differences in the residuals, a diffuse residual heatmap can correspond to differences in DLMO estimates of 15 min or more (see example in Figure 4, Panel C and sub-section *The residual heatmap* below for details).

### R Package

*dlmoR* is an R package developed to provide a streamlined and efficient tool for analyzing DLMO. The package is designed to handle both individual and batch processing of data profiles and offers a range of customizable features to meet diverse user needs.

#### Key Functionalities of the *dlmoR* Package

The core functionalities of the *dlmoR* package are:

● *Data visualization*: Generates detailed plots that include the following features (see Figure 4):
  – The raw data profile
  – The subset of points analyzed
  – The identified DLMO timestamp (labeled on the plot and output as an annotation)
  – The two segments of the hockey-stick fit with annotations of their corresponding parameters
  – A time segment highlighting the ROI for inflection point detection
  – A residual heatmap for points within the ROI grid, providing insight into the confidence or definitiveness of the result
  – A parallelogram indicating the decision boundary used to trim the points of melatonin rise, offering a clear understanding of how the subset of rise points for the fit was determined
● *Customizability*: Allows users to:
  – Set the ascending threshold
  – Set the multi-rise interval length
  – Modify plot features for tailored visualization
● *Batch processing*: Supports the analysis of multiple data profiles in a single run, enhancing efficiency for large data sets.
● *Automated Outputs*: Automatically saves all key results, including:
  – The raw data profile
  – Labeled segments
  – Fit parameters
  – DLMO point
  – Residual heatmap
  – The generated visualization plot

#### The Residual Heatmap

The residual heatmap spans the full ROI explored during the incremental search for the DLMO—the point of lowest model-fit residual. Both its bounds and values are algorithmically determined. The range is bounded below by the lowest melatonin value in the profile and above by the threshold value, while the domain bounds are defined relative to the labeled regions of the melatonin profile (*baseline, intermediate, ascending*). These regional labels are applied according to the rules described by Danilenko et al. (2014) in the original hockey-stick algorithm and depend on both the threshold value and the inter-point slopes of melatonin concentrations in the time profile. The values in the heatmap represent the residual of the hockey-stick model fit at each point in the grid. Because the heatmap directly represents this residual search space, it cannot be arbitrarily modified by the user. Instead, it serves as a diagnostic visualization of how the algorithm evaluates candidate DLMO points within the ROI.

**Figure 6.**
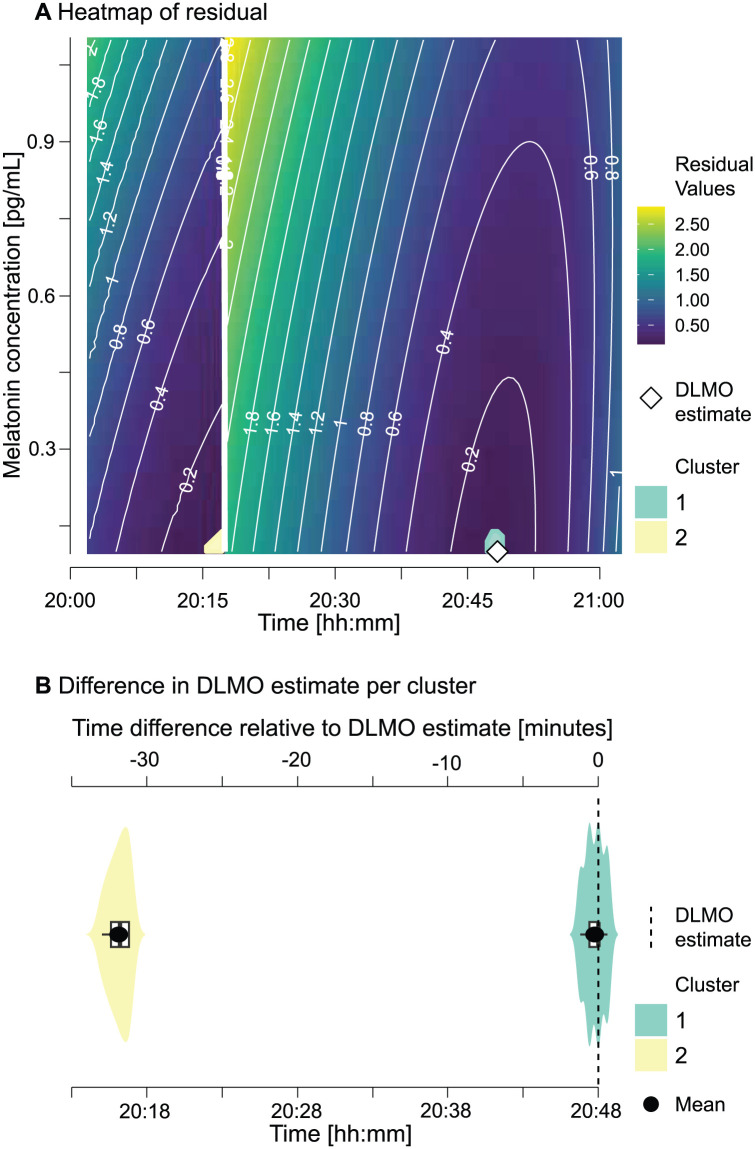
Residual landscape of candidate DLMO estimates. **a** Heatmap of hockey-stick model fit residuals for all grid points in the region of interest (ROI) for an example melatonin profile, with colors indicating residual value and contour lines tracing residual magnitude across the landscape. In this example, colored clusters mark grid points within the 0.4% lowest residuals. The *dlmoR* estimate (white diamond), corresponding to the point with the lowest residual, lies within cluster 1, while cluster 2, occurring earlier in time, contains residual values of similar magnitude, illustrating that multiple low-residual regions can exist. **b** Clock times of candidate estimates in each cluster as violin plots relative to the DLMO estimate.

While *hockeystickexe* produces a single DLMO estimate that gives the impression of an absolute, definitive solution, *dlmoR* exposes this underlying residual landscape and thereby uncovers a plurality of plausible solutions. Sharp, isolated minima indicate a well-constrained estimate, whereas diffuse or multi-modal minima reveal that the solution is embedded within a landscape of alternatives rather than being singular or definitive. In practice, many candidate estimates can yield very similar residuals while differing substantially in clock time. For instance, in the example showcased in Figure 6, the top 0.4% of residuals (31 out of 10,220 grid points) span only 0.008 in residual value (0.123–0.131) yet correspond to DLMO estimates 31.8 min apart.

To make these landscapes accessible, we developed a Shiny app for interactive diagnostics in *dlmoR* (Supplementary section D (online), Figure S2). The *Residual Heatmap Explorer* extends static residual heatmaps into an interactive tool, allowing examination of clusters of good model fit and assessment of alternative minima through distributional and statistical summaries. This interactivity reveals the hidden multiplicity of solutions, clarifying how sharply or diffusely candidate estimates are defined and generalizing the illustrative example in Figure 6 into a reusable diagnostic.

*DLMO estimate confidence intervals*: In addition to the qualitative lens offered by the heatmap exploration, *dlmoR* provides quantitative tools to formalize uncertainty assessment. We have implemented four complementary bootstrap methods for inference of DLMO confidence intervals: (1) *Monte Carlo bootstrap*, which adds Gaussian jitter to sampling times and melatonin concentrations to simulate experimental such as protocol delays and assay noise; (2) *residual bootstrap*, which resamples model residuals to test robustness of the fitted hockey-stick model; (3) *wild bootstrap*, which preserves heteroscedasticity through random multipliers, making it suitable for data sets with uneven measurement precision; and (4) *hybrid bootstrap*, which combines time jittering with residual resampling, capturing both external noise and model-fit variability in a single procedure. Each method generates an empirical distribution of DLMO estimates, summarized by its mean and an empirical 95% confidence interval. In addition, a histogram of bootstrapped DLMO estimates is generated, annotated with the original estimate, the bootstrap mean and the 95% confidence interval bounds.

Together with the residual heatmaps and the Shiny app, these bootstraps ensure that *dlmoR* does not obscure uncertainty behind a single estimate but instead makes visible and quantifiable the landscape of plausible alternatives. A comparison of the different bootstrap methods, the noise sources they simulate, and the typical use cases for each is provided in Supplementary section E.1 (online), Table S2. Example outputs from each bootstrap method are shown in Figure S3 of Supplementary section E.2 (online).

To illustrate how *dlmoR* extends and enhances the original *hockeystickexe* implementation, we provide a side-by-side comparison in Supplementary section F (online), Table S3. The table outlines key differences in platform, reproducibility, diagnostics, uncertainty quantification, and extensibility. In particular, it highlights the advances provided by *dlmoR*, including (1) open-source, cross-platform implementation in R; (2) full parameter customization (e.g. threshold levels, base slope limits, multi-rise interval lengths); (3) customizable plotting, batch processing, and scripting support; (4) reproducible workflows with transparent implementation of the hockey-stick algorithm; and two analytical functionalities not available in the original executable: (5) visualization of model-fit residual heatmaps with an accompanying Shiny app for interactive exploration; and (6) bootstrap-based methods for formal uncertainty quantification of DLMO estimates.

### In-Depth Investigation of Hockey-Stick Algorithm Behavior

Having demonstrated that *dlmoR* sufficiently concords with the *hockeystick.exe* implementation, we next leveraged its flexibility to examine the robustness and sensitivity of DLMO estimates under diverse conditions. By programmatically modifying both analysis parameters and melatonin time series characteristics, we conducted five sets of analyses designed to probe how robustly the hockey-stick algorithm recovers the DLMO timing across different scenarios. In particular, we investigated the effects of:

- **Varying sampling frequency**: to assess how changes in temporal resolution and the number of available data points influence the DLMO estimate.
- **Altering threshold values**: to determine how different choices of melatonin threshold affect the resulting DLMO estimate.
- **Irregular sampling and noise perturbations**: comprising (1) single-point deletions, (2) multi-point deletions, and (3) added noise to melatonin concentration or sample timing, to test robustness under realistic data imperfections.

We expand on the rationale, methods, and results of each analysis in the sections below.

#### Computational Environment

All computational analyses detailed below were performed either on a MacBook Pro with Apple M4 Max (12-core CPU, 38-core GPU, 16-core Neural Engine) and 36 GB unified memory, or on the NYX high-performance computing (HPC) cluster at the Max Planck Institute for Biological Cybernetics. Each NYX compute node is equipped with two AMD EPYC 7452 32-core processors (2.35 GHz; 128 logical CPUs total), and jobs were run with exclusive node access. Wall-clock times and corresponding core-hour usage for the analyses are reported in Supplementary section G (online).

#### Sampling Frequency

Rationale: This analysis investigates the sensitivity of DLMO estimates to temporal resolution of the melatonin time series, considering both dense and sparse sampling schemes. Reduced sampling rates are particularly relevant in applied research settings, where melatonin assays are costly and frequent sampling entails substantial logistical effort. Understanding how DLMO estimates behave under sparse sampling can inform practical study design decisions.

#### Methods

To quantify this effect, we performed a resampling analysis on the Blume et al. (2024) and Heinrichs and Spitschan (2025) data sets. For each melatonin profile, we used *dlmoR* to generate a reference DLMO estimate. We then resampled the profile using piecewise cubic Hermite interpolation (PCHIP), which preserves the shape and monotonicity of the original signal while avoiding spurious oscillations. Resampled time series were generated at intervals of 2, 5, 10, 15, 20, 30, 45, 60, 75, and 90 min, simulating progressively coarser sampling schemes. DLMO was re-estimated at each interval, and differences from the reference DLMO were computed to assess sensitivity to temporal resolution.

For the Blume et al. (2024) data set, sampling times were assigned based on the nominal 30-min protocol schedule, and the original DLMO estimates were calculated using these idealized timestamps. In contrast, the Heinrichs and Spitschan (2025) data set included actual recorded sample times, which varied slightly from the nominal 45-min protocol due to real-world procedural deviations. As a result, the reference DLMO estimate in Heinrichs and Spitschan (2025) did not align exactly with any fixed resampling interval.

#### Results

**Figure 7.**
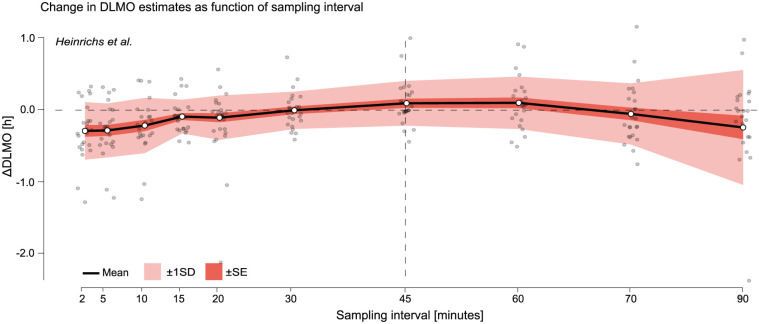
Change in DLMO estimates (in decimal hours) as a function of sampling interval for the Heinrichs and Spitschan (2025) data set. Black line shows the mean across all melatonin profiles of the difference between the DLMO estimated at each sampling interval and the reference DLMO, calculated from the actual recorded sample times, which deviated from the nominal 45-min protocol. Shaded regions indicate variability across participants: dark shading shows ±1 standard error (SE), and lighter shading shows ±1 standard deviation (SD). Gray points represent individual melatonin profiles at each resampled interval. The vertical dashed line marks the nominal 45-min sampling interval of the study protocol.

**Figure 8.**
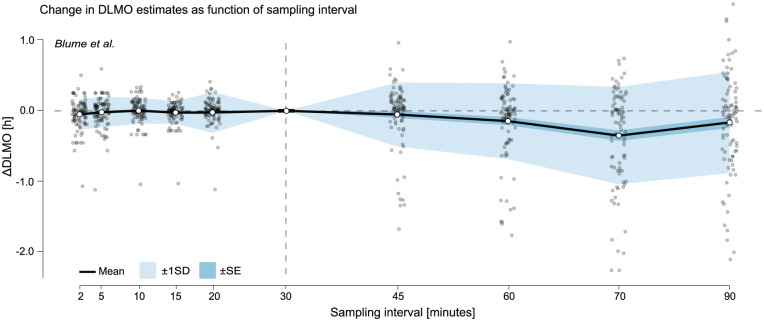
Change in DLMO estimates (in decimal hours) as a function of sampling interval for the Blume et al. (2024) data set. Black line shows the mean across all melatonin profiles of the difference between the DLMO estimated at each sampling interval and the reference DLMO, calculated from the actual recorded sample times, which followed the nominal 30-min protocol. Shaded regions indicate variability across participants: dark shading shows ±1 standard error (SE), and lighter shading shows ±1 standard deviation (SD). Gray points represent individual melatonin profiles at each resampled interval. The vertical dashed line marks the nominal 30-min sampling interval of the original protocol.

Across both Heinrichs and Spitschan (2025) and Blume et al. (2024) data sets, DLMO estimates exhibited varying sensitivity to sampling interval, with differences in both magnitude and pattern (see Figures 7 and 8 as well as Supplementary section H.1 (online) for Tables S6 and S7).

In the Heinrichs and Spitschan (2025) data set, both the mean deviation and standard deviation (SD) were elevated at short intervals (e.g. at 2-min: mean ∆DLMO =−0.2522 ± 0.3540 h), then decreased at moderate sampling intervals (15–60 min), reaching a minimum SD of 0.2295 h at 30-min sampling (see Figure 7 and Supplementary section H.1 (online), Table S6). At coarser intervals (75 and 90 min), both mean deviation and SD increased again, with SD rising to 0.3765 h and 0.7073 h, respectively. This non-monotonic pattern, with elevated variability at both fine and coarse intervals and lowest variability at intermediate resolutions (e.g. 30–45 min), suggests that the relationship between sampling interval and DLMO stability is not strictly linear in this data set.

Despite the study’s nominal 45-min sampling interval, the deviation at 45-min sampling interval was not zero (mean ∆DLMO = 0.0877 h). This reflects that the original DLMO estimates were based on the actual sample timestamps, which varied slightly due to protocol time deviations.

In the Blume et al. (2024) data set, the mean deviation remained close to zero for intervals up to 30 min (e.g. mean ∆DLMO at 20–min = 0.0226 h), and SD stayed ≤0.2853 h (see Figure 8 and Supplementary section H.1 (online), Table S7). At 30 min, no deviation was observed across all profiles because the data set used nominal timestamps based on the protocol’s fixed 30-min sampling schedule, which aligned exactly with the 30-min resampling interval. At longer intervals (60–90 min), variability and downward bias increased, with the largest mean shift at 75 min (–0.3486 h) and SD peaking at 90-min sampling intervals (0.7119 h). Note that for this data set, some resampled profiles did not yield a DLMO estimate at certain intervals, as they remained entirely sub-threshold or supra-threshold and therefore lacked the threshold crossing required by the hockey-stick algorithm.

Overall, DLMO estimates were relatively stable for intervals of ≤30 min, while intervals of ≥60 min introduced substantial variability and, in some cases, systematic bias in the mean DLMO time.

#### Threshold Values

##### Rationale

Another key factor in DLMO estimation is the choice of melatonin threshold. The hockey-stick algorithm requires defining a concentration that marks the transition from baseline to rise, yet no consensus exists on which value to use. Because thresholds are often chosen arbitrarily or vary between studies, it is important to quantify how sensitive DLMO estimates are to this parameter.

##### Methods

DLMO was estimated for each profile at thresholds of 2, 3, 4, 5, and 10 pg/mL, and shifts in DLMO timing were calculated relative to the 2 pg/mL estimate.

##### Results

**Figure 9.**
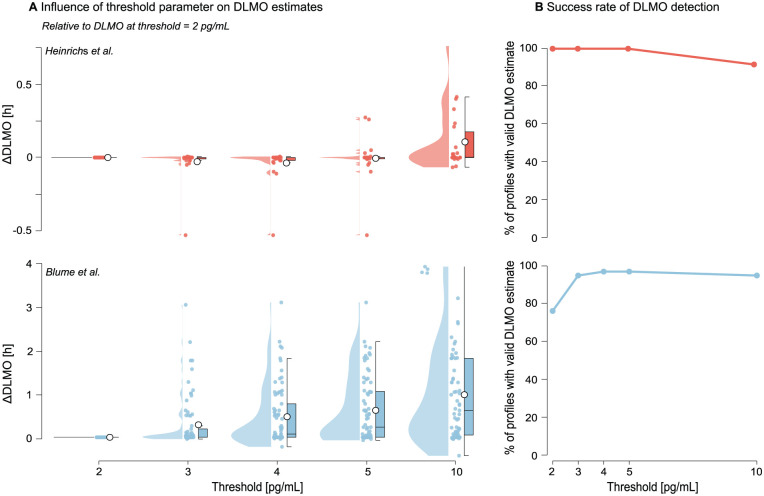
Effect of melatonin concentration threshold value on DLMO estimates and detection success for the Heinrichs and Spitschan (2025) (red) and Blume et al. (2024) (blue) data sets. a Change in DLMO estimates (in decimal hours) at each threshold, relative to the estimate at a 2 pg/mL threshold. Points represent individual profiles, with boxplots indicating the median and interquartile range and violins showing the distribution. Higher thresholds generally delayed the estimated DLMO, with larger shifts in the Blume et al. (2024) data set. b The proportion of profiles yielding a valid DLMO estimate at each threshold, with failures occurring when all melatonin values remained either below or above the threshold.

Increasing the threshold systematically delayed the estimated DLMO, with progressively larger shifts, relative to the 2 pg/mL estimate, at higher thresholds (see Figure 9 and Supplementary section H.2 (online) for Tables S10 and S11). In the Blume et al. (2024) data set, mean shifts increased from 0.2847 h at 3 pg/mL to 0.97110 h at 10 pg/mL, with variability (SD) rising from 0.5856 h to 1.1041 h. In contrast, the Heinrichs and Spitschan (2025) data set showed smaller overall shifts (e.g. mean = 0.1067 h ± 0.2048 h at 10 pg/mL). Lower thresholds (2–4 pg/mL) produced estimates that were more closely aligned, with SDs remaining below 0.12 h.

The number of profiles yielding a valid DLMO estimate varied by threshold value (see Tables 8 and 9). In both data sets, failed estimates occurred when melatonin concentrations remained entirely sub- or supra-thresholds. All 24 profiles in the Heinrichs and Spitschan (2025) data set produced valid estimates at thresholds up to 5 pg/mL, with two profiles excluded at 10 pg/mL. In contrast, the Blume et al. (2024) data set showed success rates ranging from 76% at 2 pg/mL to ≥95% at thresholds of 3 pg/mL and above.

Note that all ∆DLMO values in Figure 9 are expressed relative to the DLMO at 2 pg/mL, so the maximum number of comparable profiles in Supplementary section H.2 (online), Tables S10 and S11 is capped by the number of profiles with a valid estimate at the 2 pg/mL threshold. As a result the $n_{compared}$ values in those tables are lower than the corresponding counts *n* in the success tables (Tables S8 and S9).

To assess the robustness of DLMO estimates to missing or unreliable data, we performed a series of perturbation analyses designed to simulate common deviations from ideal sampling conditions. These included (1) single-point deletions, (2) multiple-point deletions, and (3) the addition of noise to either the melatonin concentration, the sampling time, or both. Together, these scenarios reflect challenges frequently encountered in real-world data collection, such as forgotten or missed samples, delays in experimental protocols, and intra-assay variability.

#### Single-Point Deletions

##### Rationale

Missed or invalid samples are not uncommon in melatonin sampling protocols and may affect the stability of DLMO estimation. To assess how sensitive estimates are to localized data loss, we evaluated the effect of systematically deleting individual samples.

##### Methods

For each melatonin profile, a baseline DLMO was estimated from the complete time series. Then, each data point was systematically deleted, one at a time, and the DLMO was re-estimated for each iteration. The resulting shift in DLMO (perturbed minus baseline) was recorded and associated with the timing of the deleted sample, expressed relative to the baseline DLMO. These relative deletion times were then grouped into 15-min bins, and the mean and standard deviation of the DLMO shift within each bin were computed across all profiles. Results were visualized using a ribbon plot and heatmap to highlight zones of stability and sensitivity in the melatonin curve. Although deletion effects were computed across the full extent of each profile (e.g. from −1065 to +570 min relative to baseline DLMO in Heinrichs and Spitschan (2025) and −375 to 345 min in Blume et al. (2024)), we limited visualizations to the [–120, +120] min range for visual clarity, as deviations outside this window were negligible.

#### Results

**Figure 10.**
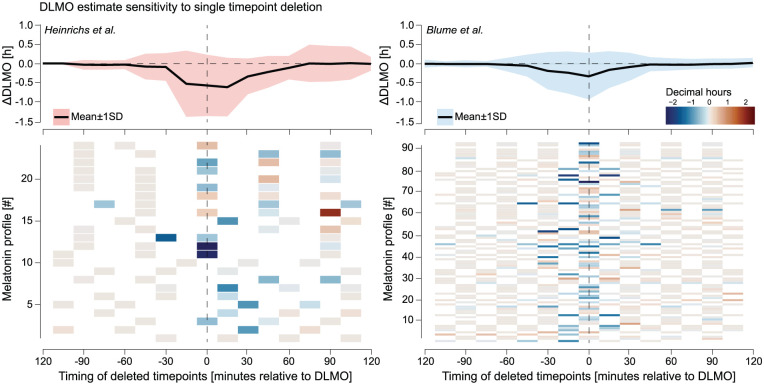
Sensitivity of DLMO estimates to single timepoint deletion for the Heinrichs and Spitschan (2025) (left, red) and Blume et al. (2024) (right, blue) data sets. For each profile, the DLMO shift resulting from deleting each timepoint was computed relative to the baseline DLMO, with deletion times expressed in minutes from that baseline. Top panels Mean (black line) ± 1 SD (shaded area) of these shifts across all profiles, binned in 15-min intervals. Bottom panels Per-profile heatmaps of the DLMO shift, with warm colors indicating later and cool colors earlier estimates. In both data sets, a narrow window within approximately ±30 min of DLMO exhibited the greatest sensitivity to deletion, while timepoints further away had little influence.

Aggregated across profiles for each data set, the analysis revealed a critical window of heightened sensitivity within approximately ±30 min of the estimated DLMO, where deleting a single timepoint produced the largest shifts in estimated timing (see Figure 10). In contrast, deletions during the stable base or late-rise phases had minimal effect.

In the Heinrichs and Spitschan (2025) data set, DLMO estimates were most sensitive to deletions within a window spanning approximately −15 to +60 min relative to the baseline DLMO estimate. The largest mean deviation occurred at +15 min ( −0.6161±0.7720 h), followed closely by deletions centered around the baseline DLMO estimate (0 min bin: −0.5706±0.8094 h) and at −15 min ( −0.5265±0.8724 h). Deletions further from DLMO, such as in the baseline phase (–120 to −60 min), had negligible influence, with mean changes typically under ±0.030 h and SD below 0.130 h. Sensitivity declined again beyond +60 min, though moderate variability persisted (SD = 0.180–0.490 h).

In the Blume et al. (2024) data set, sensitivity peaked slightly earlier, with the greatest mean deviation at 0 min ( −0.3323±0.6135 h), followed by −15 min ( −0.2380±0.5563 h) and −30 min ( −0.1901±0.4930 h). Deletions before −60 min or after +60 min had little impact, with mean shifts below ±0.030 h and SD consistently under 0.140 h (e.g. −120 min: mean = −0.0003±0.1078 h; +120 min: mean = +0.0249±0.1227 h).

The ribbon plots and heatmaps in Figure 10 illustrate these dynamics across individual profiles, revealing how a small subset of samples, especially those near the DLMO inflection point, can disproportionately affect the final estimate.

### Multi-Point Deletions

Rationale Multiple missed samples are a common occurrence in melatonin studies, arising from protocol deviations, participant noncompliance, or technical assay failures. To assess the impact of more extensive data loss, we simulated scenarios in which multiple samples were deleted.

Methods For each melatonin profile, a baseline DLMO was estimated from the complete time series. Perturbation replicates were then generated by randomly deleting 10%, 20%, 30%, 40%, or 50% of the original samples. Each deletion level was repeated 20 times per profile to account for variability in deletion patterns (see Supplementary section I (online), Figure S4 for example profile deletions). DLMO was re-estimated for each replicate, and the deviation from the baseline estimate (∆DLMO) was recorded.

#### Results

**Figure 11.**
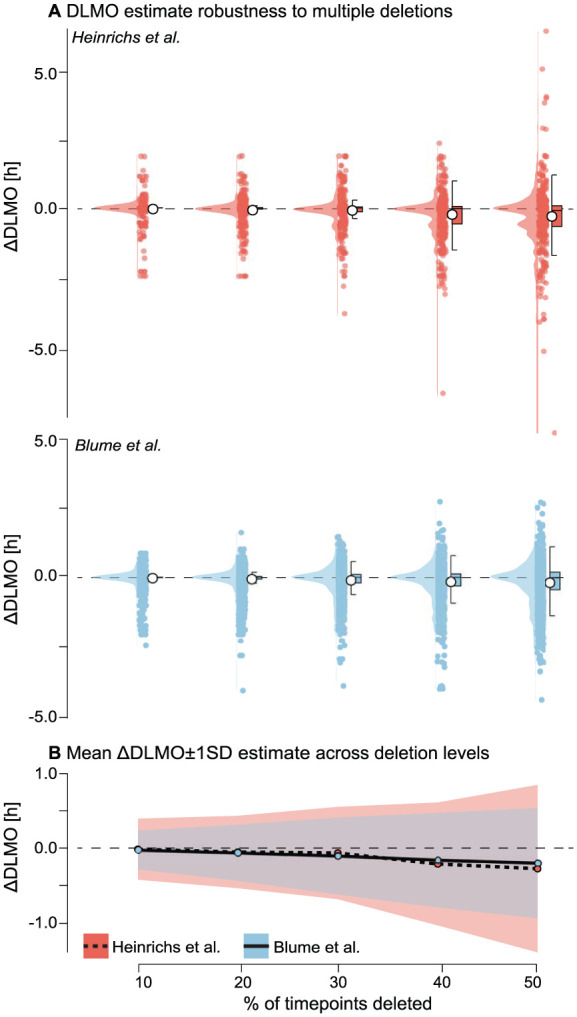
Robustness of DLMO estimates to multiple timepoint deletions for the Heinrichs and Spitschan (2025) (red) and Blume et al. (2024) (blue) data sets. a Distribution of ∆DLMO values (difference from baseline) across 20 replicate deletions at each deletion level, with points representing individual replicates, boxplots indicating median and interquartile range, and violins showing the distribution. **b** Mean ∆DLMO (line) ±1 SD (shading) across deletion levels for each data set. In both data sets, greater proportions of deleted samples led to larger deviations and greater variability, with stability at low deletion levels and marked increases above 40% deletion.

As the proportion of deleted samples increased, both data sets showed progressively larger deviations in estimated DLMO and greater variability across replicates (see Figure 11 and Supplementary section H.3 (online) for Tables S12 and S13). In the Heinrichs and Spitschan (2025) data set, the average DLMO shift grew from −0.0409±0.4094 h at 10% deletion to −0.3022±1.1233 h at 50% deletion. Estimates remained stable at lower deletion levels, with >99% of replicates yielding valid DLMO estimates up to 40% deletion. However, at 50% deletion, the success rate dropped slightly to 97.1%, indicating occasional loss of DLMO detectability.

In the Blume et al. (2024) data set, DLMO estimates were initially more stable, with mean shifts of −0.0264 h±0.2612 h at 10% deletion. But sensitivity increased at higher deletion levels, reaching an average deviation of −0.2045±0.7533 h at 50% deletion. Here, the drop in estimation success was more pronounced, decreasing from 95.6% at 10% deletion to 90.8% at 50%. In both data sets, deletions that prevented a DLMO estimate occurred when all remaining timepoints after deletion were either sub-threshold or supra-threshold, precluding a threshold crossing required by the hockey-stick algorithm.

Across profiles, the shift in estimated DLMO increased gradually with deletion percentage, with variability widening as more samples were removed. At 40% data loss, average deviations exceeded 30 min, indicating substantial instability. These patterns are visualized in violin plots, which show the full distribution of ∆DLMO across replicates, and summary plots showing the mean DLMO shift with standard deviation across replicates (see Figure 11).

### Noise Perturbations

#### Rationale

Experimental data are rarely noise-free: imprecise sampling times, participant nonadherence, and intra-assay measurement error can all perturb melatonin profiles. Such noise may affect the stability of DLMO estimation, yet its tolerance bounds remain poorly defined. To quantify robustness under these conditions, we introduced controlled perturbations along both the temporal and concentration axes of the melatonin signal.

#### Methods

Each melatonin profile was processed under six conditions: (1) a clean, noise-free baseline; (2–4) temporal jitter applied by adding Gaussian noise to sampling times, with standard deviations of 5, 10, or 20 min; (5) melatonin-only noise was modeled as multiplicative Gaussian noise with a coefficient of variation (CV) of 7.9%, corresponding to the intra-assay precision of the RK-DSM2 radioimmunoassay (NovoLytiX GmbH, Switzerland), such that the standard deviation at each timepoint scaled with melatonin concentration; (6) combined melatonin noise (CV = 7.9%) and 10-min temporal jitter. For each condition, 20 replicate profiles were generated by perturbing the original time series and interpolating values using monotonic cubic Hermite interpolation (PCHIP) to preserve signal shape. Negative values introduced by interpolation or noise were deemed physiologically implausible and were clipped to zero, with the number of occurrences recorded. DLMO was re-estimated for each replicate, and deviations from the clean baseline estimate were recorded as ∆DLMO.

#### Results

**Figure 12.**
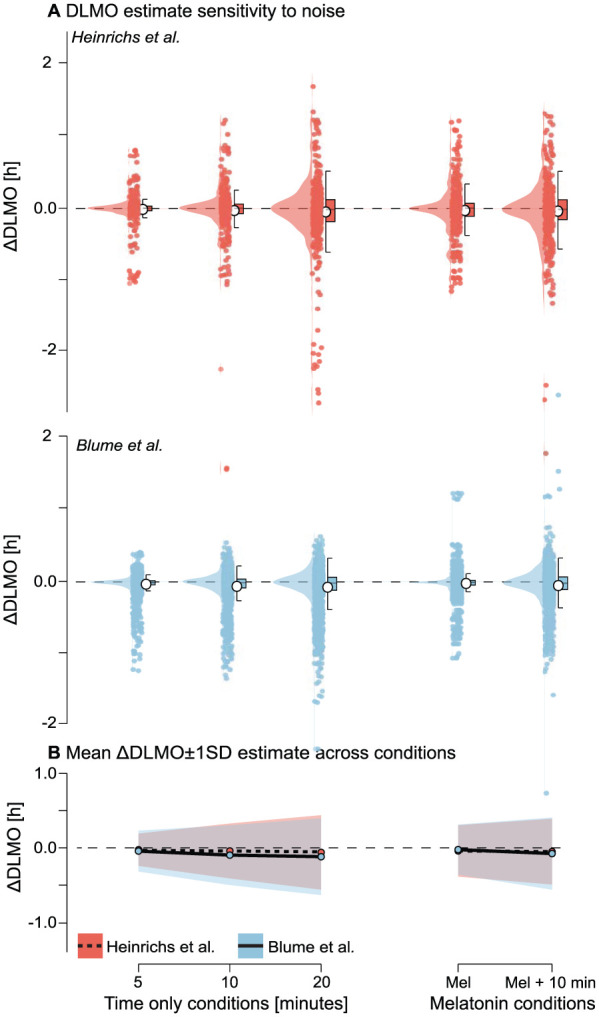
Sensitivity of DLMO estimates to noise perturbations for the Heinrichs and Spitschan (2025) (red) and Blume et al. (2024) (blue) data sets. a Distribution of ∆DLMO values (difference from the noise-free baseline) for each perturbation condition: temporal jitter applied as Gaussian noise to sampling times with different standard deviations; melatonin-only noise applied as multiplicative Gaussian noise with a coefficient of variation (CV) of 7.9% of the concentration; and combined melatonin +10-min jitter noise. Points represent individual replicates, boxplots indicate median and interquartile range, and violins show the distribution across 20 replicates per profile. b Mean ∆DLMO (line) ±1 SD (shading) for each noise condition. Across both data sets, DLMO estimates remained largely stable, with variability increasing systematically with noise magnitude and timing jitter having greater impact than melatonin-only noise. Occasional failures to produce a valid DLMO estimate in the Blume et al. (2024) occurred when all perturbed melatonin values were above or below threshold.

Across both data sets, noise perturbations introduced modest shifts in estimated DLMO, with deviations increasing systematically with noise magnitude and type (see Figure 12 and Supplementary section H.4 (online) for Tables S14 and S15). The mean absolute DLMO deviation remained below 0.0500 h for all perturbation types in the Heinrichs and Spitschan (2025) data set and below 0.1250 h in the Blume et al. (2024) data set. Variability, however, increased notably with noise level. For instance, in the Heinrichs and Spitschan (2025) data, standard deviation increased from 0.2137 h under 5-min jitter to 0.4940 h under 20-min jitter. A similar trend was observed in the Blume et al. (2024) data set, where variability rose from 0.2745 h to 0.5155 h across the same conditions.

Perturbations that affected only melatonin values (CV = 7.9%) had less impact than timing jitter: melatonin-only noise produced a mean DLMO shift of −0.0303±0.3410 h in the Heinrichs and Spitschan (2025) data set, and −0.0273±0.34 h in the Blume et al. (2024) data set. When both melatonin and timing noise were combined (10-min jitter + 7.9% CV), variability further increased (SD = 0.4363 for Heinrichs and Spitschan (2025), 0.4849 for Blume et al. (2024)), and the mean shift grew slightly more negative (–0.0396 and −0.0809 h, respectively).

All 480 runs from the Heinrichs and Spitschan (2025) data set yielded valid DLMO estimates under all perturbation conditions. In the Blume et al. (2024) data set, 10 profiles failed to produce estimates in one or more conditions, resulting in fewer runs than the full 1920 possible per condition. As in previous analyses, these failures occurred when melatonin concentrations remained entirely sub-threshold or supra-threshold. Noise perturbations and interpolation introduced negative melatonin values in 216 instances for the Heinrichs and Spitschan (2025) data set and 88 instances for the Blume et al. (2024) data set; these were subsequently clipped to zero.

## Discussion

### Concordance Between hockeystickexe and dlmoR

The high concordance between *dlmoR* and *hockeystickexe*, supported by high circular correlations and non-significant paired *t*-tests, demonstrates robust agreement. Descriptive statistics showed small mean differences, with greater variability in the Blume et al. (2024) data set than in Heinrichs and Spitschan (2025). Time-threshold assessments further indicated that most DLMO estimates were within 15 min of each other. The slight tendency for *dlmoR* to produce earlier estimates, also seen in skewness analysis, likely reflects minor numerical differences in the optimization procedure. Because the closed-source *hockeystickexe* uses undisclosed algorithms and initialization parameters, *dlmoR* applies an informed but independently selected optimization method and initial values. In addition, the hockey-stick algorithm derives the DLMO estimate from the single lowest residual within the ROI heatmap; in some analyzed profiles, hundreds of residuals differed by less than 1 ×10^−2^ in magnitude. In such cases, even the smallest difference between implementations, such as the exact start and end points of the grid search locations, can change the time point that corresponds the lowest residual, leading to substantial jumps in the estimated DLMO time. These differences are purely technical and do not involve changes to the underlying algorithm structure.

The closed-source hockey-stick implementation Danilenko and Verevkin (2020) has itself been shown to produce DLMO estimates that are, on average, 3.6±8.0 min earlier than expert judgments (see validation on page 5 in Danilenko et al. (2014)). Whether such small offsets are biologically meaningful depends substantially on context and research question: in clinical or chronotherapeutic applications, an hour’s difference could affect treatment timing, and in mechanistic studies with high statistical power, even small shifts may be critical. In contrast, population-level chronobiological research is generally less sensitive to small systematic biases if the method is consistent. For perspective, weekday–weekend DLMO differences in adolescents and young adults are typically 30–60 min with corresponding changes in sleep timing (Crowley et al., 2024; Zerbini et al., 2021), and naturalistic light exposure can advance DLMO by approximately 2 h (Wright et al., 2013). The <5–min average difference we observed between the *dlmoR* and *hockeystickexe* implementations is therefore likely biologically inconsequential. A definitive evaluation would require a well-defined ground truth, which does not currently exist.

Identified outliers in DLMO estimates between the two methods, underscore the importance of careful result interpretation and the need for users to pre-assess raw data for spurious melatonin patterns before analysis. Beyond replicating the *hockeystickexe* implementation, *dlmoR* provides an interactive Shiny-based diagnostic tool for exploring the residual heatmap of the hockey-stick fit and a function for bootstrapped confidence interval estimation. Together, these features facilitate both qualitative and quantitative assessment of DLMO uncertainty and offer insight into the robustness of each estimate.

### Hockey-Stick Algorithm Performance Under Varying Conditions

Our open-source implementation of the hockey-stick algorithm enabled us to conduct a series of analyses that probed the internal mechanics of the method by challenging it with controlled changes to sampling, thresholds, data availability, and noise. This approach revealed how specific alterations propagate through the algorithm’s region-of-interest definition and piecewise fit, sometimes producing subtle shifts in DLMO timing and in other cases causing marked deviations or failed estimates. The results set the stage for understanding why certain aspects of study design and data quality exert disproportionate influence on the DLMO outcome.

The resampling analysis showed that the hockey-stick algorithm is sensitive to both overly fine and overly coarse sampling. In the Heinrichs and Spitschan (2025) data set, variability followed a non-monotonic pattern, with elevated deviations at very short intervals (2–10 min) and at coarse intervals (≥75 min), and minimum variability at intermediate intervals (30–45 min). In contrast, the Blume et al. (2024) data set remained stable up to 30 min but showed increasing bias and variability at coarser intervals. This likely reflects differences in the original sampling schedules: the Heinrichs and Spitschan (2025) profiles were collected at intervals close to 45 min, so re-sampling to finer grids amplified the influence of small variations in timing and concentration, whereas the Blume et al. (2024) profiles were sampled every 30 min, so undersampling more quickly reduced temporal resolution and potentially masked the true onset time. Oversampling can magnify the influence of noise, while undersampling widens the gaps between points, making the DLMO search less constrained and thus broadening the domain of possible DLMO estimate locations (see *threshold analysis* below for a detailed discussion of how ROI boundaries affect the search). Our results suggest that intervals in the range of 20–45 min may provide an optimal balance between temporal precision and feasibility for this algorithm.

Raising the melatonin threshold from 2 to 10 pg/mL systematically delayed the DLMO and, at higher thresholds, increased variability. This behavior is expected because the hockey-stick algorithm uses the threshold level in two critical ways: (1) it sets the upper bound of the ROI, and (2) it determines the classification of points into *baseline*, *intermediate*, or *ascending* regions, which in turn defines the temporal limits of the ROI. Changing the threshold therefore alters both the width (i.e. time range) and height (i.e. concentration range) of the search region, as well as the subset of points available for the piecewise-linear fit, thereby directly influencing the estimated break point. The Heinrichs and Spitschan (2025) data set was notably more resistant to threshold changes than Blume et al. (2024), likely due to the steeper melatonin rise in its profiles. Larger vertical concentration changes between consecutive samples make the baseline–ascending boundary (and thus the ROI limits) less sensitive to small threshold shifts.

Given the strong influence of threshold level on DLMO timing, this choice should be made transparently and, where possible, applied consistently across samples and studies to enable comparability. In most cases, a uniform threshold is recommended; however, in rare instances, individual melatonin profiles may require an alternative, for example, when concentrations never approach the standard value or when unusually high baseline levels obscure the onset. Our recommendations for choosing and reporting a standard threshold level, and handling of exceptional cases, are provided in Supplementary section J (online).

The single-point deletion analysis revealed a narrow window of heightened sensitivity spanning just before to shortly after the onset of melatonin rise. Within this range, removing a single sample could shift the estimate by 30–40 min or more. This behavior reflects the algorithm’s dependence on ROI boundaries: the ROI begins at the last *baseline* point, includes any *intermediate* points, and extends into the *ascending phase.* Deleting a point that marks one of these transition bounds alters the ROI limits, which in turn redefines the grid search domain and the set of candidate DLMO estimates. By contrast, deleting points well outside this transition zone affects the piecewise-linear fits for individual segments but leaves the ROI bounds intact, thereby limiting their influence on the final DLMO estimate.

Multi-point deletions compounded these effects. While removing 10–20% of samples produced limited shifts in the DLMO estimate, deletions exceeding 30% caused substantial instability, with mean deviations exceeding 30 min at 40% loss. Deletions affect the estimate both by widening the ROI (due to greater gaps between the remaining points) and by reducing the number of points available to fit each segment, making the break point more susceptible to shifts in position. Profiles with shallow melatonin rises or low peak concentrations were especially vulnerable, and in some cases failed to yield a valid estimate when the profile became entirely sub- or supra-threshold.

Perturbations mimicking temporal jitter and assay noise revealed that the hockey-stick algorithm is particularly susceptible to changes in sample timing. This is because assignment of region labels to points, and thus ROI bounds, depend on sample times and slopes. Small timing shifts can change the classification of points from *baseline* to *intermediate* or even *ascending* (or vice versa), which moves the ROI start earlier or later and alters the search domain. While melatonin concentration noise alone had a smaller effect, the combination of timing jitter and amplitude noise amplified both bias and variability, and in some cases prevented estimation altogether by pushing all points into sub- or supra-threshold ranges.

This in-depth stress-test analysis of the hockey-stick algorithm demonstrates that its performance is shaped in systematic but uneven ways by analytical choices and common data imperfections. In doing so, it provides a framework for understanding the robustness, limitations, and optimal use of the hockey-stick algorithm across diverse experimental contexts. By deliberately perturbing sampling schedules, threshold levels, data completeness, and signal fidelity, we identified both the conditions under which DLMO estimates remain stable and the scenarios that introduce substantial bias or prevent estimation altogether. Its reliance on a fixed threshold, explicit ROI bounds, and a piecewise-linear fit makes this algorithm sensitive to sampling frequency, threshold selection, data loss, and sampling error. To mitigate these vulnerabilities, we recommend that researchers intending to use the hockey-stick algorithm for DLMO estimation: (1) select sampling intervals that capture the rising phase with sufficient resolution; (2) pre-specify and justify the threshold concentration; (3) minimize missing data in the ±1 h window around the expected DLMO; and (4) apply quality checks to flag profiles at risk of failed estimation and, where necessary, adjust parameters accordingly. Implementing such safeguards can improve reproducibility and ensure that DLMO estimates reflect accurate onset times within realistic data quality constraints.

### Future Directions in DLMO Estimation

The development of the hockey-stick algorithm by Danilenko et al. (2014) was a key step forward in providing an objective framework for determining DLMO, addressing the subjectivity inherent in earlier approaches. However, upon closer examination, this method is not as definitively objective as it might initially appear. The sensitivity of its optimization process, particularly to initialization and local minima, reveals underlying challenges, especially in complex tasks like the multi-constrained optimization of the parallelogram used to select the subset of ascending-phase points for fitting with the hockey-stick model. This sensitivity, coupled with the diffuse nature of the ROI heatmap, highlights that the solutions derived are not singular or definitive but instead represent a range of plausible possibilities. Rather than masking this inherent uncertainty, we propose a shift toward approaches that maintain objectivity while transparently representing the spread of potential solutions. The diffuse heatmaps often found in our profile analyses suggest that the DLMO is not a precise timestamp but rather a region of highest likelihood.

One promising refinement within the same general class of piecewise-linear models is the use of the *segmented* package in R (R News, 2008), which implements the approach described by Muggeo (2003) for estimating breakpoints and their uncertainty in regression models. Unlike the discrete search grids or fixed candidate points used in the hockey-stick algorithm, *segmented* treats breakpoints as model parameters, iteratively estimating them alongside regression slopes using a score-based method (Fasola et al., 2018). This framework supports formal statistical inference, including standard errors and confidence intervals for breakpoints, with more recent advances improving interval estimation through smoothed score-based approaches (Muggeo, 2017). By allowing multiple breakpoints, varying slopes before and after the onset, and hypothesis testing to evaluate the significance of added breakpoints, *segmented* addresses a key limitation of the traditional hockey-stick algorithm: the lack of formal statistical inference and uncertainty quantification for the estimated onset point. While still grounded in the conceptual simplicity of piecewise-linear transitions, *segmented* offers a more rigorous framework for breakpoint estimation, making it better suited for data sets where the rise in melatonin is gradual or noisy.

Despite these advantages, *segmented*, like other breakpoint-based methods, ultimately produces a single best estimate of the onset point, supplemented by a confidence interval. This still frames DLMO as a fixed timestamp rather than a distribution over possible values. A fully probabilistic approach, such as a Gaussian process model (GPM) (Rasmussen and Williams, 2006), could better capture this uncertainty, modeling DLMO as a probabilistic distribution over a plausible range rather than a single optimal point. GPMs offer several advantages in this context. By representing melatonin profiles as smooth, non-linear functions, they provide flexibility and adapt directly to the data without imposing rigid assumptions like breakpoints. They also naturally account for uncertainty by providing credible intervals for potential DLMO regions, offering a more transparent and nuanced view of melatonin onset. In addition, GPMs avoid the pitfalls of optimization-based methods, such as sensitivity to initialization or local minima, by calculating distributions over plausible solutions, making them particularly well-suited for noisy or complex data sets.

Alternatively, generalized additive mixed models (GAMMs) (Pedersen et al., 2019) provide another flexible, data-driven approach for estimating DLMO. Unlike Gaussian process models, which take a fully probabilistic approach, GAMMs model melatonin changes as smooth, continuous curves that adapt to the underlying patterns in the data while incorporating fixed and random effects. GAMMs are particularly well-suited to capturing gradual or complex trends without relying on abrupt transitions, such as breakpoints in piecewise models. They also account for individual variability through random effects, allowing the model to adjust for differences across individuals, such as chronotype variation, while still identifying shared patterns. In addition, GAMMs can pinpoint the sharpest increase in melatonin levels using derivatives of the smooth function, providing a precise and objective estimate of DLMO without relying on pre-defined thresholds.

As we continue to develop the *dlmoR* package, we aim to build it into a modular suite of DLMO estimation methods, enabling users to select, apply, and directly compare multiple approaches within a single framework. Such flexibility would support both methodological transparency and the exploration of how different modeling assumptions influence DLMO estimates.

Equally important to expanding methodological breadth is ensuring the reproducibility and accessibility of these tools. Reproducibility is a cornerstone of scientific research, enabling findings to be validated and extended by others (Harrison et al., 2021). Yet many scientific software tools lack the transparency, accessibility, and adaptability necessary to meet these standards, hindering broader adoption and integration into diverse research workflows (Wilson et al., 2017; Harrison et al., 2021). Our open-source R package addresses these challenges by promoting reproducible large-scale analyses and facilitating community-driven contributions. Unlike existing standalone .exe software, which limits modifiability and flexibility, this package adheres to the principles of findability, accessibility, interoperability, and reusability (FAIR), fostering an ecosystem for collaborative development and innovation (Lee et al., 2021; Wilson et al., 2017). Through modular design, clear documentation, version control, and continuous integration, *dlmoR* is designed for both immediate usability and long-term sustainability. By releasing it openly, we aim to equip researchers with a robust, extensible platform for melatonin analysis and a foundation for future methodological advances.

## Conclusion

In this article, we introduced the *dlmoR* package, an open-source R implementation of the hockey-stick algorithm (Danilenko et al., 2014) licensed under the MIT license. Our implementation reproduces the results of the original closed-source executable while making the algorithm transparent and extensible. Through a series of systematic stress tests, we used *dlmoR* to evaluate the robustness of the hockey-stick algorithm, showing that its performance depends in uneven but interpretable ways on sampling schedules, threshold choice, data completeness, and signal fidelity. These analyses provide practical guidance for use of the hockey-stick method for DLMO estimation across experimental contexts. The *dlmoR* package thus establishes a reproducible and sustainable foundation for melatonin-based circadian phase estimation and a modular platform for incorporating future quantitative approaches to DLMO detection.

## Supplemental Material

sj-pdf-1-jbr-10.1177_07487304251389994 – Supplemental material for dlmoR: An Open-Source R Package for the Dim-Light Melatonin Onset (DLMO) Hockey-Stick Method Supplemental material, sj-pdf-1-jbr-10.1177_07487304251389994 for dlmoR: An Open-Source R Package for the Dim-Light Melatonin Onset (DLMO) Hockey-Stick Method by Salma M. Thalji and Manuel Spitschan in Journal of Biological Rhythms

## Notes

1. Supplementary material is available for this article online. *Supplementary section A:* Pseudocode of the hockey-stick algorithm implemented in *dlmoR* *Supplementary section B:* Hockey-stick algorithm parameter rationale *Supplementary section C:* Visualization of outlier cases *Supplementary section D:* Residual heatmap explorer Shiny app *Supplementary section E:* Bootstrapping methods in *dlmoR* *Supplementary section F:* Comparison of *dlmoR* and *hockeystickexe* features *Supplementary section G:* Computational runtime of hockey-stick algorithm analyses *Supplementary section H:* Analysis summary tables *Supplementary section I:* Example profiles for multiple deletion analysis *Supplementary section J:* Guidance on choosing a threshold value for DLMO estimation

2. The description of this portion of the algorithm in the publication by Danilenko et al. (2014) differed from the natural-language explanation provided in personal correspondence, in regards to the use of weighting for the L2-norms of the fits based on base labels. In our *dlmoR* implementation, we chose not to apply any weighting, and after conducting multiple trials, we found no evidence that weighting improved the results.
