# Supplementary material for: dlmoR: An Open-Source R Package for the Dim-Light Melatonin Onset (DLMO) Hockey-Stick Method
Source: J Biol Rhythms. 2026 Feb 12;41(3):301–23. doi: 10.1177/07487304251389994 (PMC13103346; doi:10.1177/07487304251389994)
Supplement: sj-pdf-1-jbr-10.1177_07487304251389994 – Supplemental material for dlmoR: An Open-Source R Package for the Dim-Light Melatonin Onset (DLMO) Hockey-Stick Method [file sj-pdf-1-jbr-10.1177_07487304251389994.pdf]

Supplementary material

dlmoR: An open-source R package for the dim-light melatonin onset (DLMO) hockey-stick method

Journal of Biological Rhythms, December 2025

Salma M. Thalji[1,2] and Manuel Spitschan[1,2,3]

[1] Translational Sensory & Circadian Neuroscience, Max Planck Institute for Biological Cybernetics, Tübingen, Germany
[2] Chronobiology & Health, TUM School of Medicine and Health, Technical University of Munich, Munich, Germany
[3] TUM Institute for Advanced Study (TUM-IAS), Technical University of Munich, Garching, Germany

Overview of contents:

Supplementary section A: Pseudocode of the hockey-stick algorithm implemented in *dlmoR*
Supplementary section B: Hockey-stick algorithm parameter rationale
Supplementary section C: Visualization of outlier cases
Supplementary section D: Residual heatmap explorer Shiny app
Supplementary section E: Bootstrapping methods in dlmoR
Supplementary section F: Comparison of *dlmoR* and *hockeystickexe* features
Supplementary section G: Computational runtime of hockey-stick algorithm analyses
Supplementary section H: Analysis summary tables
Supplementary section I: Example profiles for multiple deletion analysis
Supplementary section J: Guidance on choosing a threshold value for DLMO estimation

Supplementary section A: Pseudocode of the hockey-stick algorithm implemented in dlmoR

Algorithm 1: Define *base* region of the melatonin profile

Input: Time series data containing melatonin concentrations $M[i]$, timestamps $t[i]$ (in POSIXct format), and a threshold value τ (default = 2.3 pg/mL)

Output: Annotated time series with base segment labeled $B[i]$ (1 for base, 0 for non-base)

Step 1: Preprocess Data;

Sort the time series data chronologically;

Convert timestamps from POSIXct format to decimal hours to create a continuous numerical representation of time;

Step 2: Compute Profile Slopes;

Calculate the slope between consecutive data points. The slope at point i is defined as:

$$\text{slope}[i] = \frac{M[i] - M[i-1]}{t[i] - t[i-1]},$$

where $M[i]$ is the melatonin concentration and $t[i]$ is the time in decimal hours.

Step 3: Identify Base Segments;

For each point in the time series:

- Label as **base** (`1`) if:

  $$\text{slope}[i] \leq 0 \quad \text{and} \quad \big(M[i] \leq \tau \text{ or } M[i+1] \leq \tau\big),$$

  where $M[i+1]$ is the concentration at the subsequent point.

- Otherwise, label as **non-base** (`0`).

Step 4: Propagate Base Labels;

Propagate the base segment label (`1`) backward from the rightmost identified base point. A point i is labeled as base if:

$$B[i] = \begin{cases} 1 & \text{if } i \leq \max\{j \mid B[j] = 1\}, \\ 0 & \text{otherwise.} \end{cases}$$

This ensures that all points preceding the last base point are also labeled as base.

Algorithm 2: Define *ascending* region of the melatonin profile

Input: Time series data containing melatonin concentrations $M[i]$, timestamps $t[i]$, slopes $\text{slope}[i]$, and *base* labels; threshold value τ (default = 2.3 pg/mL); interval limit Δt_{limit} (default = 2 hours)

Output: Annotated time series with ascending segments labeled $A[i]$ (1 for ascending, 0 for non-ascending)

Indexing and initialization:;

Assume i is 1-indexed and $t[1] < t[2] < \cdots < t[n]$. At $i = 1$, define:

$$T[1] = \begin{cases} 1 & \text{if } M[1] > \tau, \\ 0 & \text{otherwise.} \end{cases}$$

Initialize:

$$G[1] = \begin{cases} 1 & \text{if } T[1] = 1, \\ \text{NA} & \text{if } T[1] = 0. \end{cases}$$

Step 1: Detect Transitions Above Threshold;

For $i \geq 2$, identify transitions where melatonin concentrations rise above threshold τ after being below or equal to it:

$$T[i] = \begin{cases} 1 & \text{if } M[i] > \tau \text{ and } M[i-1] \leq \tau, \\ 0 & \text{otherwise.} \end{cases}$$

These transitions mark the starting points of potential ascending segments.

Step 2: Group Consecutive Points into Rise Groups;

For $i \geq 2$, assign:

$$G[i] = \begin{cases} G[i-1] + 1 & \text{if } T[i] = 1, \\ G[i-1] & \text{if } G[i-1] \neq \text{NA}, \\ \text{NA} & \text{otherwise.} \end{cases}$$

A new group is started when a transition is detected ($T[i] = 1$), and points remain in the same group if consecutive.

Step 3: Validate Rise Groups Based on Interval Limit;

Let $i_g = \min\{i : G[i] = g\}$ be the first index in group g.

Define the group start time: $t_g = t[i_g]$.

The inter-group interval is: $\Delta t_g = t_g - t_{g-1}$ ($g \geq 2$).

A rise group g is valid if:

$$V[g] = \begin{cases} 1 & \text{if } g = 1 \text{ or } \Delta t_g \geq \Delta t_{\text{limit}}, \\ 0 & \text{otherwise.} \end{cases}$$

This ensures that only rise groups separated by a sufficient time interval are retained.

Step 4: Assign Ascending Labels;

Label points within valid rise groups as ascending if their melatonin concentration exceeds the threshold:

$$A[i] = \begin{cases} 1 & \text{if } G[i] \in \mathcal{V} \text{ and } M[i] > \tau, \text{ where } \mathcal{V} = \{g : V[g] = 1\}, \\ 0 & \text{otherwise.} \end{cases}$$

Step 5: Identify Steepest Segments;

Let $\text{slope}_{\max} = \max_i \text{slope}[i]$. Label points as steep if:

$$S[i] = \begin{cases} 1 & \text{if } \text{slope}[i] \geq \frac{1}{2} \cdot \text{slope}_{\max}, \\ 0 & \text{otherwise.} \end{cases}$$

This captures significant changes even without a corresponding rise above the threshold.

Step 6: Backward Propagation of Ascending Labels;

Propagate the ascending label backward from the first ascending point to earlier points if their slopes are sufficiently steep. For any preceding point j of the first ascending point i:

$$A[j] = \begin{cases} 1 & \text{if } \text{slope}[j] \geq \frac{1}{2} \cdot \text{slope}[i] \text{ and } A[j+1] = 1, \\ 0 & \text{otherwise.} \end{cases}$$

Stop when the slope condition is no longer satisfied or when $j = 1$.

return *Annotated time series with* $A[i]$ *(1 for ascending, 0 for non-ascending)*;

Algorithm 3: Truncate *ascending* region

Input: Time series data containing melatonin concentrations $M[i]$, timestamps $t[i]$, slopes $\text{slope}[i]$, and ascending labels $A[i]$.

Output: Updated time series with truncated ascending segment and optimal parallelogram fit parameters.

Step 1: Check for Ascending Segment;

Extract the ascending segment:

$$A_{\text{segment}} = \{i \mid A[i] = 1\}.$$

if $A_{segment}$ *is empty* **then**

  Issue a warning: "No ascending segments found in the profile data.";

  return *Input data unchanged.*

end

Step 2: Find Steepest Slope in Ascending Segment;

Compute the maximum slope in the ascending segment:

$$\text{max\_slope} = \max(\text{slope}[i] \mid i \in A_{\text{segment}}).$$

Step 3: Iteratively Truncate Ascending Segment;

Truncate the ascending segment until all rules are satisfied:

- **Rule 1: Positive rightmost slope.** The slope between the last two points in the ascending segment must be positive:

$$\text{rightmost\_slope} = \frac{M[i_{\text{last\_asc}}] - M[i_{\text{last\_asc}-1}]}{t[i_{\text{last\_asc}}] - t[i_{\text{last\_asc}-1}]} > 0.$$

- **Rule 2: Valid parallelogram fit.** Ensure the parallelogram fit (Algorithm 4) does not violate constraints, such as diagonal slope rules.

while *either rule is violated* **do**

  Identify the last ascending index:

$$i_{\text{last\_asc}} = \max\{i \mid A[i] = 1\}.$$

  Set the last point in the ascending segment to non-ascending:

$$A[i_{\text{last\_asc}}] \leftarrow 0.$$

  Recompute max_slope.

end

Step 4: Finalize Parallelogram Fit;

Fit a parallelogram to the updated profile data using Algorithm 4:

$$\text{parallelogram\_fit(profile\_data)}.$$

return *Updated profile data and parallelogram fit parameters.*

Algorithm 4: Fit optimal parallelogram to enclose points with minimal area

Input: Coordinates of points $x[i]$, $y[i]$ in the Cartesian plane.

Output: Optimal parallelogram parameters: x_0 (left edge), x_1 (right edge), and slope.

Step 1: Define Initial Variables;

Compute vertical bounds of the parallelogram:

$$y_0 = \min(y[i]), \quad y_1 = \max(y[i]).$$

Set initial guesses for optimization:

- $x_0^{\text{initial}} = x[1]$ (first point's x coordinate),
- $x_1^{\text{initial}} = x[N]$ (last point's x coordinate),
- Slope:
$$\text{slope}^{\text{initial}} = \frac{y_1 - y_0}{x_1^{\text{initial}} - x_0^{\text{initial}}}.$$
- Adjust x_1 based on slope and vertical difference:
$$x_1^{\text{adjusted}} = x_1^{\text{initial}} - \frac{y_1 - y_0}{\text{slope}^{\text{initial}}}.$$
- Initial guess for optimization:
$$\text{initial\_guess} = \left(x_0^{\text{initial}} \cdot 0.8,\ x_1^{\text{adjusted}} \cdot 1.1,\ \text{slope}^{\text{initial}}\right).$$

Step 2: Define Parallelogram Corners;

Compute the corners of the parallelogram based on x_0, x_1, y_0, y_1, and slope:

$$\text{Corners:}\ \begin{aligned} \text{Lower Left (LL):} &\quad (x_0, y_0), \\ \text{Lower Right (LR):} &\quad (x_1, y_0), \\ \text{Upper Left (UL):} &\quad \left(x_0 + \frac{y_1 - y_0}{\text{slope}}, y_1\right), \\ \text{Upper Right (UR):} &\quad \left(x_1 + \frac{y_1 - y_0}{\text{slope}}, y_1\right). \end{aligned}$$

Step 3: Define Constraints;

Enforce constraints to ensure all points $(x[i], y[i])$ lie within the parallelogram:

$$\begin{aligned} c_{\text{upper}}[i] &= y_1 + \text{slope} \cdot (x[i] - x_1) - y[i], \\ c_{\text{lower}}[i] &= y[i] - \left(y_0 + \text{slope} \cdot (x[i] - x_0)\right), \\ c_{\text{left}}[i] &= x[i] - \min(x_0, x_0 + \frac{y_1 - y_0}{\text{slope}}), \\ c_{\text{right}}[i] &= \max(x_1, x_1 + \frac{y_1 - y_0}{\text{slope}}) - x[i]. \end{aligned}$$

Compute the constraint penalty as:

$$c_{\text{penalty}} = \min(c_{\text{upper}}, c_{\text{lower}}, c_{\text{left}}, c_{\text{right}}).$$

Step 4: Define Objective Function;

Minimize the parallelogram area while penalizing constraint violations:

$$\text{Objective: area} + \lambda \cdot \log(1 + c_{\text{penalty}}^2),$$

where:

$$\text{area} = \left|x_1 - x_0\right| \cdot \left|y_1 - y_0\right|.$$

Step 5: Perform Optimization;

Optimize x_0, x_1, and slope using the initial guess:

$$\text{result} = \text{optim(initial\_guess, objective\_function, method = "SANN")}.$$

Step 6: Return Optimal Parameters;

Extract and return optimal parameters:

$$x_0^{\text{opt}},\ x_1^{\text{opt}}, \text{slope}^{\text{opt}}.$$

Algorithm 5: Add a segment to the left of the *ascending* region

Input: Time series data containing melatonin concentrations $M[i]$, timestamps $t[i]$, ascending labels $A[i]$, and threshold value τ.

Output: Updated time series with a new segment added on the left, if necessary.

Step 1: Check if All Segments Belong to the Ascending Region;

if *all points satisfy* $A[i] = 1$ **then**

Identify the leftmost node in the ascending segment:

$$i_{\text{leftmost}} = \min\{i \mid A[i] = 1\}.$$

Let the corresponding melatonin concentration and timestamp be:

$$M_{\text{leftmost}} = M[i_{\text{leftmost}}], \quad t_{\text{leftmost}} = t[i_{\text{leftmost}}].$$

end

Step 2: Check Threshold Condition for the Leftmost Node;

if $M_{\textit{leftmost}} < 0.5 \cdot \tau$ **then**

Compute the melatonin concentration for the new node:

$$M_{\text{new}} = \max\left(0, \frac{M_{\text{leftmost}}}{2}\right).$$

Compute the timestamp for the new node:

$$t_{\text{new}} = t_{\text{leftmost}} - 30 \text{ minutes}.$$

end

Step 3: Add the New Segment;

Create a new node with:

$$t_{\text{new}},\ M_{\text{new}},\ A_{\text{new}} = 0,\ B_{\text{new}} = 0,\ I_{\text{new}} = 1.$$

Append the new node to the time series.

Step 4: Return Updated Time Series;

return *Time series with the added intermediate segment.*

Algorithm 6: Truncate *base* region based on threshold

Input: Time series data containing melatonin concentrations $M[i]$, timestamps $t[i]$, and base labels $B[i]$; threshold value τ (default = 2.3 pg/mL).

Output: Updated time series with truncated base segment.

Step 1: Preprocess Data;

Sort the time series data chronologically by timestamps:

$$t[1] \leq t[2] \leq \cdots \leq t[N].$$

Step 2: Identify Base Nodes Above Threshold;

For each point i in the base segment ($B[i] = 1$):

- Set $B[i] = 0$ if:

$$M[i] > \tau \quad \text{and} \quad \sum_{j=1}^{i} \mathbb{1}(B[j] = 1 \text{ and } M[j] \leq \tau) = 0.$$

- This condition ensures that all base nodes exceeding the threshold at the start of the base segment are truncated.

Step 3: Check for Remaining Base Segment;

if *all remaining points have* $B[i] = 0$ **then**

Issue a warning: "Hockey-stick time: no base part.";

return *Input data with base set to 0.*

end

Step 4: Return Updated Data;

return *Time series with truncated base segment labels* $B[i]$.

Algorithm 7: Define *intermediate* segment in a melatonin profile

Input: Time series data containing melatonin concentrations $M[i]$, timestamps $t[i]$, base labels $B[i]$, and ascending labels $A[i]$; threshold value τ.

Output: Annotated time series with intermediate segments labeled $I[i]$ (1 for intermediate, 0 otherwise).

Step 1: Preprocess Data;

Sort the time series data chronologically by timestamps;

Identify the indices of base and ascending segments:

- **Last base row:**
$$i_{\text{last\_base}} = \max\{i : B[i] = 1\}.$$
- **First ascending row:**
$$i_{\text{first\_asc}} = \min\{i : A[i] = 1\}.$$

Step 2: Check for Intermediate Rows;

If $i_{\text{last\_base}} < i_{\text{first\_asc}} - 1$:

- Identify rows between the last base and first ascending:
$$I[j] = 1 \quad \text{for } j \in (i_{\text{last\_base}}, i_{\text{first\_asc}}).$$
- Otherwise, set $I[j] = 0$ for all j.

Step 3: Handle Special Cases;

- **Case 1: No base points exist.**
  - If all rows before $i_{\text{first\_asc}}$ have melatonin concentrations $M[j] < \min(M[i_{\text{first\_asc}}], \tau)$, label those rows as intermediate:
$$I[j] = 1 \quad \text{for } j \in \{1, \ldots, i_{\text{first\_asc}} - 1\}.$$
  - Additionally, set the first row as a base point:
$$B[1] = 1.$$
- **Case 2: All ascending points except the first row.**
  - If $i_{\text{first\_asc}} = 2$ and $B[1] = 0$, label the first point as intermediate:
$$I[1] = 1.$$
  - Create a synthetic base point 30 minutes before the first row:
$$t_{\text{new}} = t[1] - 30 \text{ minutes}, \quad M_{\text{new}} = \frac{M[1]}{2}.$$
    Insert this new row as a base point.

Step 4: Return Annotated Data;

return *Time series with updated intermediate labels* $I[i]$.

Algorithm 8: Compute region of interest (ROI) for DLMO point search

Input: Time series data with melatonin concentrations $M[i]$, timestamps $t[i]$, base labels, ascending labels, and (if available) intermediate labels; threshold value τ (default = 2.3 pg/mL).

Output: Region of interest (ROI) with bounds: $x_{\text{start}}, x_{\text{end}}, y_{\text{min}}, y_{\text{max}}$.

Step 1: Preprocess Data;

Sort the time series data chronologically by timestamps;

Identify subsets of points based on labels:

- **Base points:** Points where the base label $B[i] = 1$.
- **Ascending points:** Points where the ascending label $A[i] = 1$.
- **Intermediate points (if available):** Points where the intermediate label $I[i] = 1$.

Step 2: Determine Horizontal Bounds;

- **Case 1: Intermediate points are present.**

$$x_{\text{start}} = t_{\text{last}}^{\text{base}} + 0.1 \cdot \left(t_{\text{first}}^{\text{intermediate}} - t_{\text{last}}^{\text{base}}\right),$$
$$x_{\text{end}} = t_{\text{last}}^{\text{intermediate}} + 0.95 \cdot \left(t_{\text{first}}^{\text{ascending}} - t_{\text{last}}^{\text{intermediate}}\right).$$

- **Case 2: Fewer than two base points.**

$$x_{\text{start}} = t_{\text{first}}^{\text{base}} + 0.05 \cdot \left(t_{\text{first}}^{\text{ascending}} - t_{\text{first}}^{\text{base}}\right),$$
$$x_{\text{end}} = t_{\text{last}}^{\text{base}} + 0.95 \cdot \left(t_{\text{first}}^{\text{ascending}} - t_{\text{last}}^{\text{base}}\right).$$

- **Case 3: More than two base points are available.**

$$x_{\text{start}} = \text{mean}\left(t_{\text{last-1}}^{\text{base}}, t_{\text{last}}^{\text{base}}\right),$$
$$x_{\text{end}} = t_{\text{last}}^{\text{base}} + 0.95 \cdot \left(t_{\text{first}}^{\text{ascending}} - t_{\text{last}}^{\text{base}}\right).$$

Step 3: Determine Vertical Bounds;

- The lower bound is the minimum melatonin concentration in the profile:

$$y_{\text{min}} = \min(M[i]).$$

- The upper bound is the threshold:

$$y_{\text{max}} = \tau.$$

Step 4: Return ROI;

return *ROI with bounds:* $x_{start}, x_{end}, y_{min}, y_{max}$.

Algorithm 9: Determine DLMO point using coarse and refined grid search

Input: Time series data with melatonin concentrations $M[i]$ and timestamps $t[i]$ (in POSIXct format); threshold value τ (default = 2.3 pg/mL); region of interest (ROI); grid step sizes for coarse and refined searches (Δx, Δy, Δx_{small}, Δy_{small}); fit type (e.g., linear or parabolic).

Output: Point of inflection P_{inf} (DLMO point); parameters for base and ascending segment fits.

Step 1: Generate Coarse Grid Points;

- Define the ROI as $x \in [x_{\min}, x_{\max}]$ and $y \in [y_{\min}, y_{\max}]$.
- Generate a coarse grid of candidate points:

$$G_{\text{coarse}} = \{(x, y) : x \in \text{seq}(x_{\min}, x_{\max}, \Delta x),\ y \in \text{seq}(y_{\min}, y_{\max}, \Delta y)\}.$$

Step 2: Fit Profiles and Evaluate Residuals (Coarse Grid);

foreach *candidate point* $P = (x, y) \in G_{coarse}$ **do**

- Split the data into **base segment** (points $t[i] \leq x$) and **ascending segment** (points $t[i] > x$).
- Perform linear or parabolic fitting for both segments:

$$\text{Base fit:} \quad y_{\text{base}}[i] = m_{\text{base}} \cdot (t[i] - x) + y,$$

$$\text{Ascending fit:} \quad y_{\text{asc}}[i] = \begin{cases} m_{\text{asc}} \cdot (t[i] - x) + y & \text{(linear fit)}, \\ a \cdot t[i]^2 + b \cdot t[i] + c & \text{(parabolic fit)}. \end{cases}$$

- Compute residuals:

$$R = \frac{1}{N} \sum_i \left(M[i] - y_{\text{pred}}[i]\right)^2,$$

where $y_{\text{pred}}[i]$ is the predicted melatonin value from the base or ascending fit.

if $R < R_{best}$ **then**

Update $P_{\text{best}} \leftarrow P$, $R_{\text{best}} \leftarrow R$, and store the fit parameters.

end

end

Step 3: Refine Grid Around Best Candidate;

- Identify a reduced ROI around P_{best} by expanding the range:

$$x'_{\min} = \max(x_{\text{best}} - \Delta x, x_{\min}), \quad x'_{\max} = \min(x_{\text{best}} + \Delta x, x_{\max}),$$

$$y'_{\min} = \max(y_{\text{best}} - \Delta y, y_{\min}), \quad y'_{\max} = \min(y_{\text{best}} + \Delta y, y_{\max}).$$

- Generate a refined grid of points:

$$G_{\text{refined}} = \{(x, y) : x \in \text{seq}(x'_{\min}, x'_{\max}, \Delta x_{\text{small}}),\ y \in \text{seq}(y'_{\min}, y'_{\max}, \Delta y_{\text{small}})\}.$$

Step 4: Fit Profiles and Evaluate Residuals (Refined Grid);

Repeat Step 2 over G_{refined}, further minimizing R to determine the final inflection point P_{inf}.

Step 5: Return Results;

return *P_{inf} (point of inflection), parameters for the base fit (m_{base}) and ascending fit (m_{asc} or a, b, c), grid points, and residuals.*

Supplementary section B: Hockey-stick algorithm parameter rationale

Table S1. Overview of decision criteria used in the DLMO detection algorithm, organized by processing stage, with associated variables and physiological/algorithmic rationale.

Algorithm Stage	Variable / Criterion	Rationale
Ascending region definition — Rules 2–3	Steepness $\geq 1/2$ of the steepest segment (in non-base portion and preceding first ascending segment)	A relative steepness cut-off set at $1/2$ the maximum slope adapts to each profile's amplitude and rate of change. Applied near the start of the ascent, it avoids including slow, gradual rises that precede the main physiological melatonin increase. Because small, irregular slopes from measurement variability or short-term fluctuations are typically well below this relative threshold, they are also excluded as a side-effect.
Ascending region truncation — Rule 1	Zero or negative slope in final segment	Removes flat or falling tails from the ascending region, which cannot contribute to the rise and may bias the DLMO estimate, by altering the ascent model-fit, if retained.
Ascending region truncation — Rule 2: *Parallelogram rule*	Ratio of parallelogram's longest diagonal slope to lateral edge slope $> 1/2$	The fitted parallelogram's lateral edge defines the steepest ascent that still encloses all ascending points and the last pre-ascending point (geometric upper bound), while the longest diagonal gives the average ascent over the region. Their ratio measures how closely the actual rise approaches the maximally steep, sustained rise allowed by the data. A ratio below $1/2$ indicates a brief, disproportionately fast late segment following a slow body, which can delay DLMO estimates; these are iteratively removed until the remaining ascent is both rapid on average and consistent across its span.
Ascending region truncation — Rule 3	Final segment $\geq 1/2$ the steepness of steepest remaining segment	After Rules 1 and 2 are applied, this rule trims late segments that rise too slowly. This prevents inclusion of gentle, tapering tails that contribute little to the rise but can shift the modeled DLMO later in time.
ROI definition	Line of interest: last 50% of final base segment (no intermediate segments) or from after last base node including all intermediate segments (if present) to first 95% of first ascending segment	Defines the time span used as the horizontal bounds of the ROI, targeting the transition from baseline to ascent. When no intermediate segments are present, starting at the last 50% of the final base segment captures the baseline portion closest to onset while avoiding distant flat baseline. When intermediate segments bridge the base and ascending phases, including all of them preserves transitional shapes, such as shallow early rises, needed for accurate onset detection. In both cases, ending at 95% of the first ascending segment avoids its terminal portion, where slopes may flatten, ensuring the ROI focuses on the part of the rise most representative of DLMO onset.
Coarse phase of DLMO search	Linear fit to earlier points constrained to pass through POI; slope limited to -0.2 to 0.2	Constraining the fit to pass through the POI ensures continuity between the two halves of the profile when estimating the inflection. Limiting the slope range to [-0.2,0.2] prevents unrealistic trends in the baseline portion: it avoids overly steep negative slopes that would imply implausible declines before melatonin onset, and overly steep positive slopes that could artificially inflate the apparent rate of rise, biasing the DLMO earlier.
Refinement phase of DLMO search	Refinement-phase parabola constraints (i–iv)	Passing through the POI (i) keeps the refined fit anchored to the candidate inflection point from the coarse phase. Ensuring the derivative is never zero (ii) avoids introducing local plateaus that would blur the definition of onset. Requiring the slope at the POI to be at least $1/2$ of the slope of the coarse-phase later linear fit (iii) prevents the refined curve from being unrealistically shallow at the onset compared to the initial coarse estimate. Demanding it be steeper than the coarse-phase earlier linear fit (iv) enforces a clear contrast between baseline and ascent. Together, these constraints refine the onset location while keeping the later fit both physiologically plausible and consistent with the data.

Supplementary section C: Visualization of outlier cases

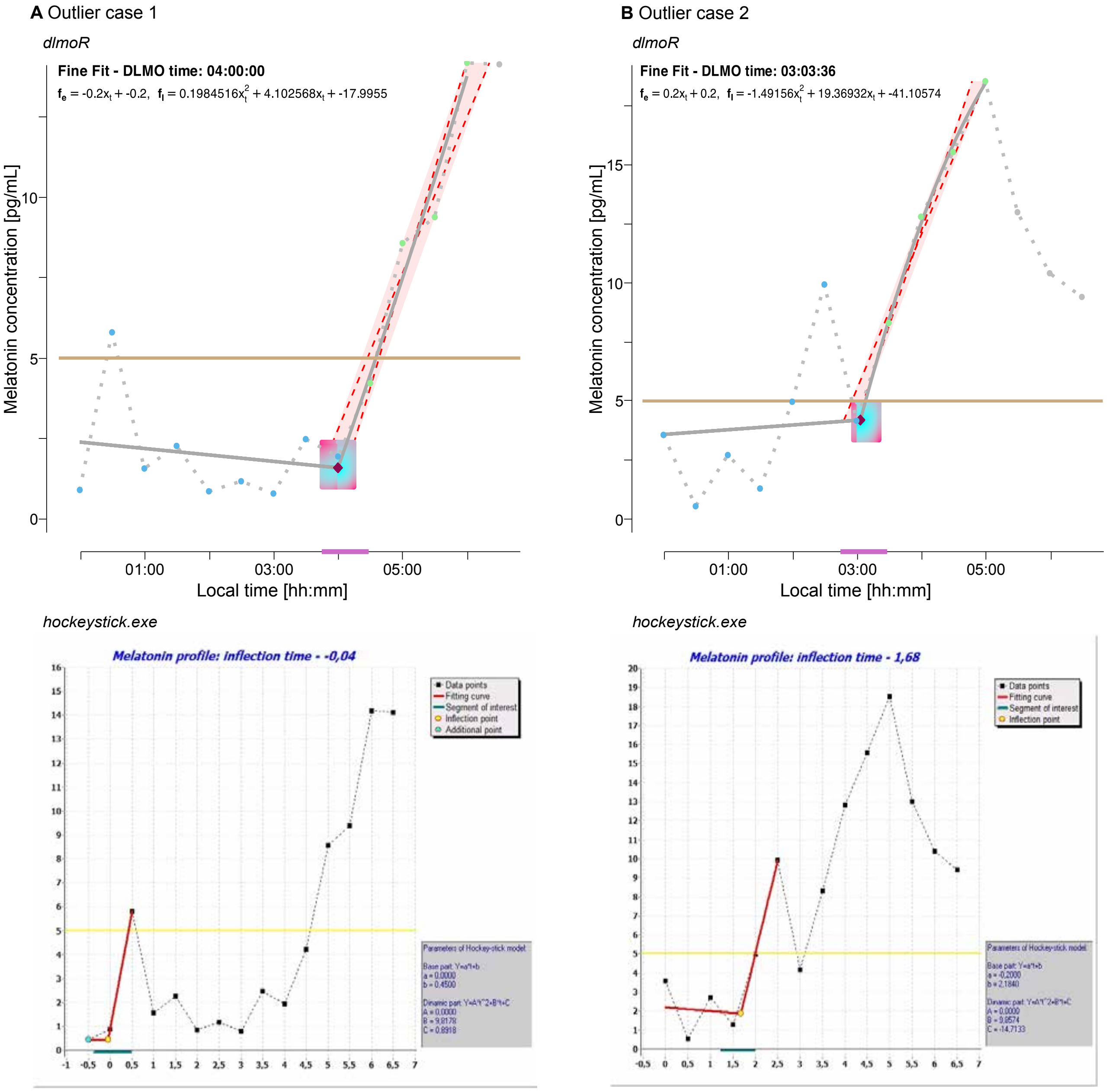

Figure S1. The only two major outlier cases among 112 profiles, showing large discrepancies between *dlmoR* (top row) and *hockeystickexe* (bottom row) DLMO estimates. **A** Outlier case 1: a single early above-threshold melatonin value was fit by *hockeystickexe*, whereas *dlmoR* fit the later sustained rise, yielding a 80-minute difference in DLMO estimates. **B** Outlier case 2: *hockeystickexe* fit a spurious early rise, while *dlmoR* identified the sustained rise 240 minutes later. **Top panels**: the dark purple point marks the DLMO estimate, solid grey lines the model fits, the ochre horizontal line the threshold, the light purple line the ROI range for the coarse fit, and shaded areas the parallelogram used to trim ascending segments. **Bottom panels**: yellow points mark DLMO estimates, solid red lines the model fits, yellow horizontal line the threshold, and the green line the ROI range for the coarse fit.

Supplementary section D: Residual heatmap explorer Shiny app

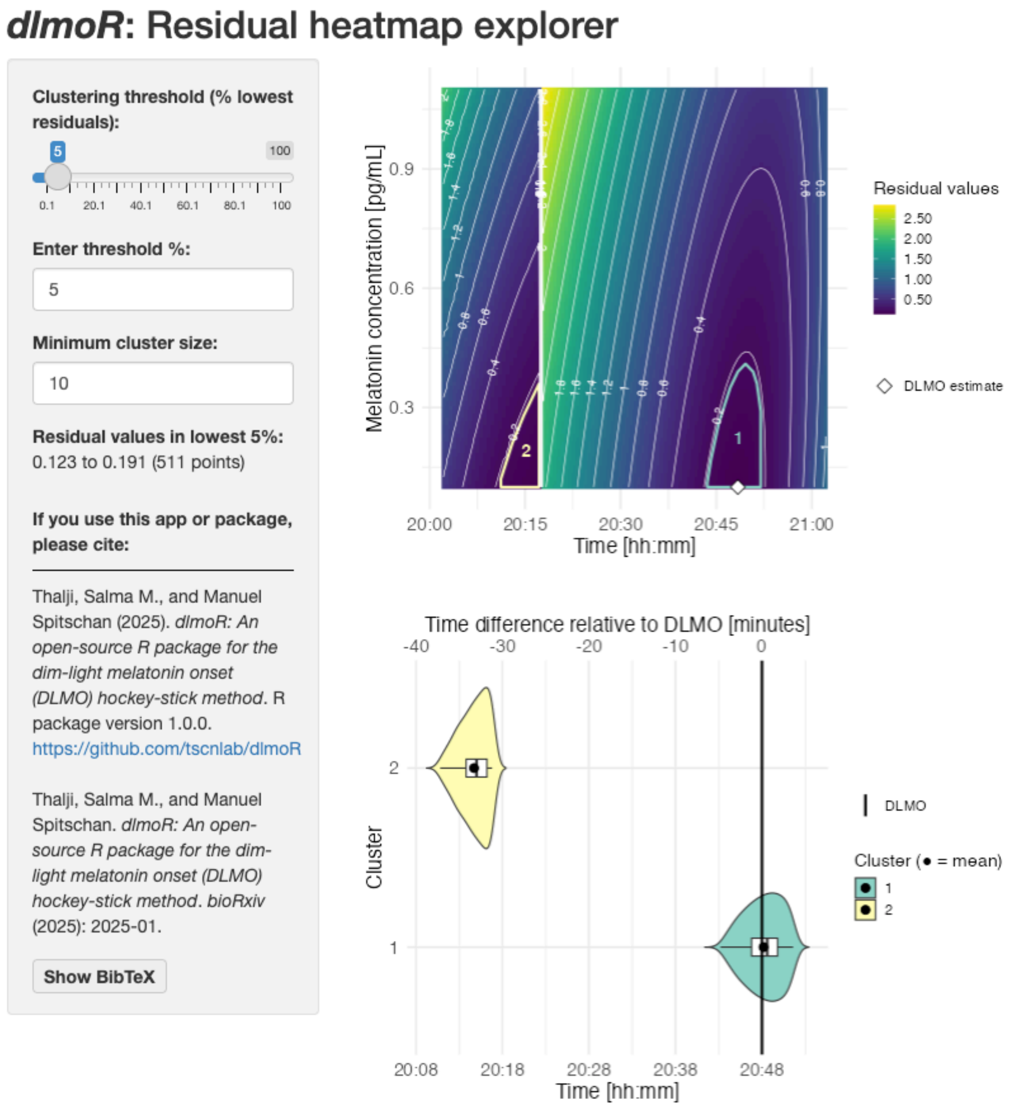

DLMO time: 20:48:23

Cluster stats: nominal

Cluster	Size	% of grid	Mean time	Median time	Min time	Max time
1	337	3.30	20:48	20:48	20:43	20:51
2	174	1.71	20:15	20:15	20:11	20:17

Cluster stats: time difference relative to DLMO

Cluster	Mean [min]	Median [min]	Min [min]	Max [min]
1	0.19	0.60	-4.80	3.60
2	-33.29	-33.00	-37.20	-31.20

Figure S2. Example output from the *dlmoR: Residual heatmap explorer* Shiny app, a *post-hoc* tool that takes the DLMO data structure output from *dlmoR* as input and visualizes the surrounding residual landscape of the ROI. It allows users to assess whether the landscape is unimodal or multimodal, and how focused or diffuse it is—offering insight into how definitive the DLMO fit may be. **Top left**: The control panel allows users to adjusts the clustering threshold—via sliding bar or numeric input—to set the percentage of lowest residual values included in clusters and the minimum cluster size. **Top right**: The heatmap shows residual values for a single melatonin profile, with contour lines indicating magnitude. Colored regions mark clusters within the specified threshold, and the white diamond marks the DLMO estimate. **Middle right** The violin plot shows candidate DLMO times per cluster, relative to the estimate. **Bottom**: Summary tables list cluster size, proportion of the grid, and timing range.

Supplementary section E: Bootstrapping methods in *dlmoR*

E.1 Comparison of DLMO estimate bootstrapping methods

Table S2. Comparison of bootstrap methods implemented in *dlmoR*.

Method	Noise source(s) simulated	Key parameters	Typical use case
**Monte Carlo**	Simulates realistic experimental variability by perturbing both sampling times and melatonin concentrations:<br>• Temporal jitter of samples<br>• Heteroscedastic assay noise proportional to concentration	• *time_sd*: SD of time jitter (default = ½ minimum sampling interval)<br>• *mel_cv*: coefficient of variation for assay noise (default = 0.079)<br>• *clip_negatives*: enforce non-negative concentrations	When protocol timing errors (late/early sampling) and assay variability substantially contribute to DLMO uncertainty; realistic simulation of experimental conditions
**Residual**	Quantifies variability due to deviations around the fitted hockey-stick model without introducing timing or assay noise:<br>• Resampling residuals from baseline and ascending fits	• *clip_negatives*: enforce non-negative concentrations	When timing and assay precision are well-controlled; isolates uncertainty from measurement scatter around the model fit
**Wild**	Generalizes the residual bootstrap by applying random weights to residuals, preserving heteroscedasticity:<br>• *rademacher*: ± 1 sign flips<br>• *normal*: Gaussian weights	• *wild_type*: residual weighting scheme<br>• *clip_negatives*: enforce non-negative concentrations	Robust uncertainty estimation under heteroscedastic residuals; *rademacher* ($w \in \{-1, +1\}$) for robustness, *normal* ($w \sim \mathcal{N}(0, 1)$) for Gaussian-like variability
**Hybrid**	Combines Monte Carlo and residual approaches to simulate both timing irregularities and residual noise:<br>• Temporal jitter of samples<br>• Residual resampling added to jittered model	• *time_sd*: SD of time jitter (default = ½ minimum sampling interval)<br>• *clip_negatives*: enforce non-negative concentrations	Conservative choice for sensitivity analysis; captures both experimental (timing) and statistical (residual) uncertainties

E.2 Example outputs of DLMO estimate variability across bootstrap schemes

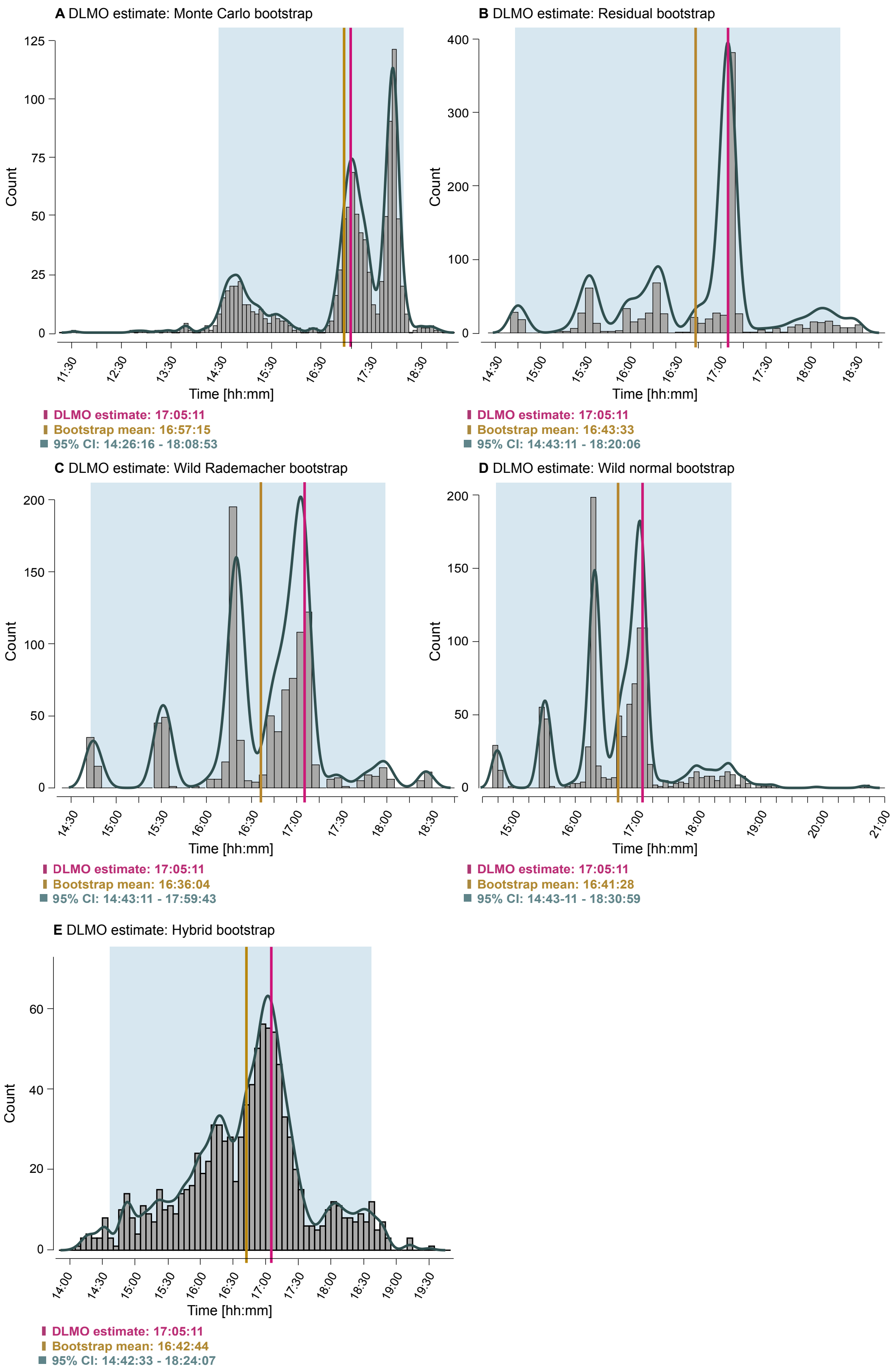

Figure S3. Example distributions of DLMO estimates obtained using five bootstrap methods implemented in dlmoR. Panels show histograms and kernel density estimates for a single melatonin profile under: **A** Monte Carlo bootstrap, which perturbs both sampling times and melatonin concentrations to simulate realistic experimental variability; **B** Residual bootstrap, which resamples model residuals to quantify uncertainty from measurement scatter; **C** Wild bootstrap with Rademacher weights and **D** Wild bootstrap with normal weights, both generalizing the residual approach by applying random weights to residuals; and **E** Hybrid bootstrap, which combines Monte Carlo and residual resampling to capture both timing and residual noise. Vertical magenta lines mark the original DLMO estimate, ochre lines the bootstrap mean, and shaded blue regions the 95% confidence intervals.

Supplementary section F: Comparison of *dlmoR* and *hockeystickexe* features

Table S3. Comparison of *dlmoR* and *hockeystickexe* feature availability

**Feature**	***hockeystickexe***	***dlmoR***
**Platform**	•Windows-only executable<br>•Requires virtualization or compatibility layer to run on macOS/Linux	•Runs natively on Windows, macOS, and Linux via R<br>•Works in local, server, and cloud environments
**License**	Closed source (unknown)	Open source (MIT License)
**Residual Heatmap**	Not provided	•Provided numerically as residual matrices (coarse and fine)<br>•Visual overlay in plots<br>•Interactive Shiny app for zooming and stats
**Estimate Confidence Intervals**	Not provided	Five bootstrapping methods for *post hoc* CI estimation
**Batch Processing**	Not supported	Fully supported via scripting
**Parameter Customization**	•Threshold<br>•Base segment slope limits	•Threshold<br>•Base segment slope limits<br>•Multi-rise interval length<br>•Optional coarse-only fitting mode<br>•Full access to algorithm internals (open source)
**Plotting**	•Fixed-format PNG<br>•No customization	•Export formats: PNG, JPEG, SVG, R object<br>•Fully customizable:<br>•Melatonin profile<br>•Model fit overlays<br>•DLMO estimate<br>•ROI and threshold lines<br>•Segment classification<br>•Residual heatmaps (coarse and fine)<br>•Trimming parallelogram overlay
**Outputs**	•DLMO in HH:MM:SS<br>•Annotated plot of fine phase fit	•DLMO in HH:MM:SS, POSIXct, decimal hours<br>•Coarse and fine model parameters<br>•Grid intercepts and residuals<br>•Full plot object for coarse and fine phases<br>•Complete data structure (fully scriptable)
**Output Saving Options**	•PNG only<br>•Fixed file path and name	•Fully scriptable saving<br>•Directory control and custom naming<br>•Multiple formats<br>•Selective component output

Supplementary section G: Computational runtime of hockey-stick alogrithm analyses

Table S4. Wall-clock time and core-hour usage for analyses of the Heinrichs and Spitschan (2025) dataset.

Analysis	*Machine*	*Wall-clock time*	*Core-hours*
Sampling interval	MacBookPro	3 hours 11 minutes	41.38
Threshold	MacBookPro	1 hours 31 minutes	19.72
Single-deletion	MacBookPro	1 hours 31 minutes	19.72
Multiple deletion	HPC Cluster	11 hours 46 minutes	282
Noise	HPC Cluster	15 hours 15 minutes	366

Table S5. Wall-clock time and core-hour usage for analyses of the Blume et al. (2024) dataset.

Analysis	*Machine*	*Wall-clock time*	*Core-hours*
Sampling interval	MacBookPro	5 hours 02 minutes	65.43
Threshold	MacBookPro	2 hours 44 minutes	35.53
Single-deletion	MacBookPro	9 hours 55 minutes	9.92
Multiple deletion	HPC Cluster	21 hours 26 minutes	1372
Noise	HPC Cluster	27 hours 14 minutes	1742

Supplementary section H: Analysis summary tables

H.1 DLMO estimate sensitivity to sampling interval

Table S6. Δ DLMO *vs* sampling interval (Heinrichs et al.)

interval [min]	*mean Δ [h]*	*SD Δ [h]*	*SE Δ [h]*	*n*
2	-0.2522	0.3540	0.0723	24
5	-0.2470	0.3317	0.0677	24
10	-0.1862	0.3402	0.0694	24
15	-0.0763	0.2077	0.0424	24
20	-0.0899	0.2730	0.0557	24
30	0.0014	0.2295	0.0468	24
45	0.0877	0.2759	0.0563	24
60	0.0931	0.3225	0.0658	24
75	-0.0435	0.3765	0.0769	24
90	-0.2103	0.7073	0.1444	24

Table S7. Δ DLMO *vs* sampling interval (Blume et al.)

interval [min]	*mean Δ [h]*	*SD Δ [h]*	*SE Δ [h]*	*n*
2	-0.0501	0.2150	0.0225	91
5	-0.0200	0.2136	0.0225	90
10	0.0025	0.1820	0.0190	92
15	-0.0250	0.1577	0.0166	90
20	-0.0226	0.2853	0.0297	92
30	0.0000	0.0000	0.0000	93
45	-0.0520	0.4496	0.0479	88
60	-0.1150	0.5322	0.0564	89
75	-0.3486	0.6837	0.0733	87
90	-0.1637	0.7119	0.0763	87

H.2 DLMO estimate sensitivity to threshold value

Table S8. DLMO estimate success per threshold (Heinrichs et al., N=24)

threshold [pg/mL]	*n*	*% success*
2	24	100.0
3	24	100.0
4	24	100.0
5	24	100.0
10	22	91.7

Table S9. DLMO estimate success per threshold (Blume et al., N=96)

threshold [pg/mL]	*n*	*% success*
2	73	76.0
3	91	94.8
4	93	96.9
5	93	96.9
10	91	94.8

Table S10. Δ DLMO (relative to threshold = 2) *vs* threshold (Heinrichs et al.)

threshold [pg/mL]	*mean Δ [h]*	*SD Δ [h]*	$n_{compared}$
2	0.0000	0.0000	24
3	-0.0282	0.1078	24
4	-0.0374	0.1115	23
5	-0.0055	0.1413	23
10	0.1067	0.2048	22

Table S11. Δ DLMO (relative to threshold = 2) *vs* threshold (Blume et al.)

threshold [pg/mL]	*mean Δ [h]*	*SD Δ [h]*	$n_{compared}$
2	0.0000	0.0000	73
3	0.2847	0.5856	73
4	0.4684	0.6782	73
5	0.6138	0.7473	72
10	0.9710	1.1041	67

H.3 DLMO estimate sensitivity to multi-point deletions

Table S12. Δ DLMO *vs* % deletion (Heinrichs et al.)

% del	*mean Δ [h]*	*SD Δ [h]*	*n*	*N*	*% success*
10	-0.0409	0.4094	480	480	100.0
20	-0.0789	0.4853	480	480	100.0
30	-0.0913	0.6187	479	480	99.8
40	-0.2384	0.8235	476	480	99.2
50	-0.3022	1.1233	466	480	97.1

Table S13. Δ DLMO *vs* % deletion (Blume et al.)

% del	*mean Δ [h]*	*SD Δ [h]*	*n*	*N*	*% success*
10	-0.0264	0.2612	1835	1920	95.6
20	-0.0645	0.3786	1829	1920	95.3
30	-0.1082	0.5183	1810	1920	94.3
40	-0.1637	0.6372	1797	1920	93.6
50	-0.2045	0.7533	1743	1920	90.8

H.4 DLMO estimate sensitivity to various sources of noise

Table S14. Δ DLMO *vs* noise condition, (Heinrichs et al.)

noise type	*mean Δ [h]*	*SD Δ [h]*	*n*	*N*
Time only: 5 min	-0.0145	0.2137	480	480
Time only: 10 min	-0.0307	0.3641	480	480
Time only: 20 min	-0.0500	0.4940	480	480
Mel only: 5 min	-0.0303	0.3410	480	480
Mel + time: 10 min	-0.0396	0.4363	480	480

Table S15. Δ DLMO *vs* noise condition (Blume et al.)

noise type	*mean Δ [h]*	*SD Δ [h]*	*n*	*N*
Time only: 5 min	-0.0470	0.2745	1830	1920
Time only: 10 min	-0.1015	0.4015	1825	1920
Time only: 20 min	-0.1232	0.5155	1810	1920
Mel only: 5 min	-0.0273	0.3366	1864	1920
Mel + time: 10 min	-0.0809	0.4849	1830	1920

Supplementary section I: Example profiles for multiple deletion analysis

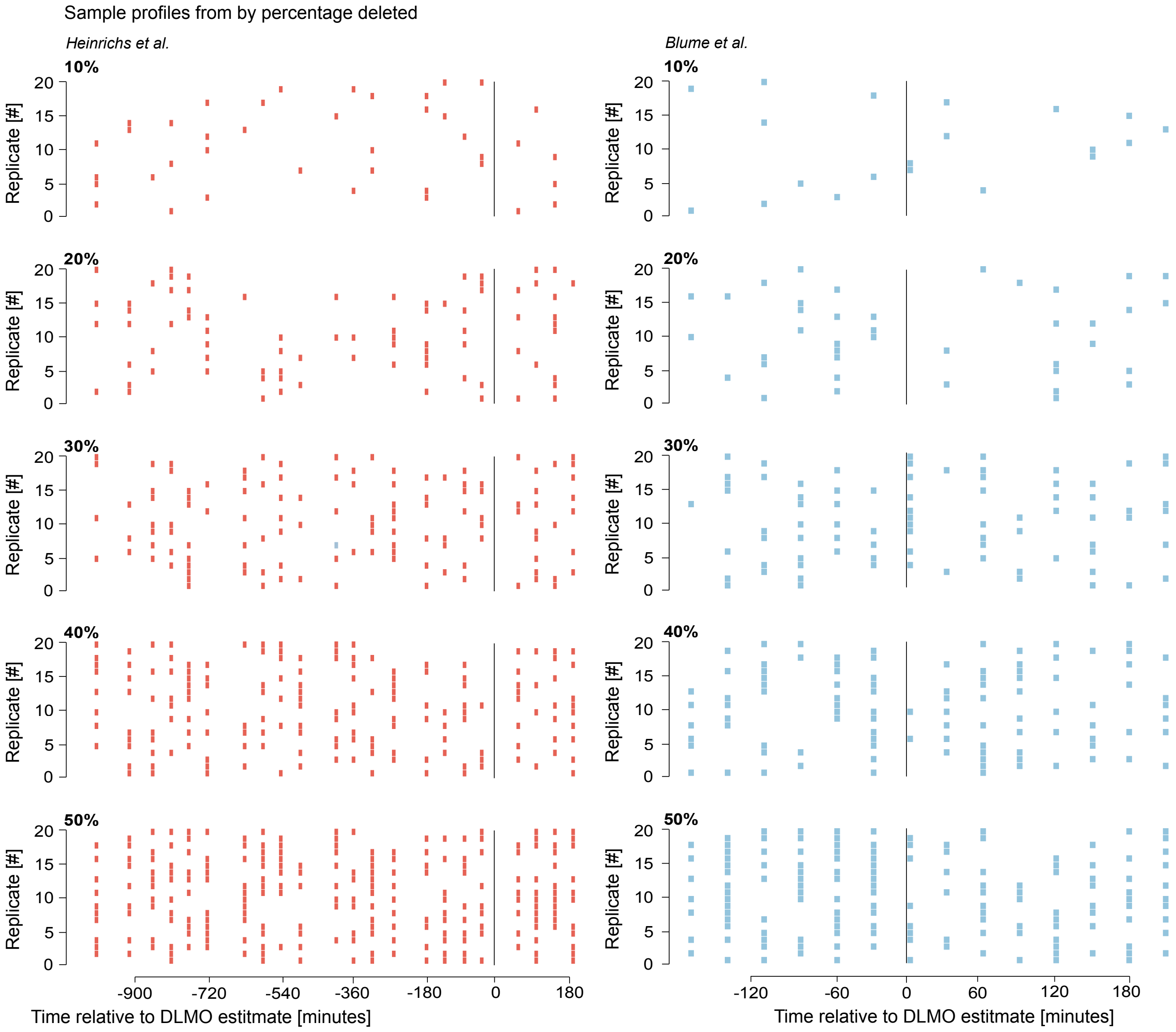

Figure S4. Example raster plots of melatonin profiles after random deletion of 10–50% of samples, for Heinrichs and Spitschan (2025) **(red, left)** and Blume et al. (2024) **(blue, right)**. Each row shows one deletion replicate (20 per profile per deletion level), with points marking the remaining samples after deletion, plotted relative to the baseline DLMO estimate from the complete, undeleted profile (vertical black line at time zero). Because deletions were applied at random within each profile, the pattern of missing samples varies between replicates, illustrating how temporal coverage of the melatonin curve is progressively reduced in different ways as the percentage of deleted samples increases. The wider time axis range in the Heinrichs and Spitschan (2025) panel reflects the longer sampling window of those profiles compared to the Blume et al. (2024) dataset. These same deletion replicates form the basis for the multi-point deletion robustness analysis shown in Section *Results: Multi-point deletions*, Fig. **??**.

Supplementary section J: Guidance on choosing a threshold value for DLMO estimation

Selecting an appropriate threshold is a critical step in estimating dim light melatonin onset (DLMO) using the hockey-stick algorithm. While there is no universally applicable threshold, we recommend the following pragmatic and transparent approach:

1. **Start with a standard threshold**
   Begin with a fixed, commonly used threshold (e.g., 3 pg/mL) that has been demonstrated to yield reliable DLMO estimates across a wide range of individuals. This approach maximizes consistency and facilitates comparison across studies.

2. **Identify problematic profiles**
   For some participants, the standard threshold may fail to yield a valid DLMO estimate. This can occur when the entire melatonin profile remains either entirely below (sub-threshold) or above (supra-threshold) the chosen value, or when the melatonin rise is too shallow or too steep to clearly define an onset point. These profiles should be flagged for individual review and may require an adaptive or individualized thresholding approach.

3. **Use a data-driven alternative for flagged cases**
   In such instances, a statistical threshold (e.g., the mean plus 2 standard deviations of the first 3 samples) (see Voultsios et al. (1997); Molina and Burgess (2011); Kennaway (2023); Murray et al. (2024), may provide a more appropriate and biologically meaningful estimate. Any deviation from the standard method should be pre-specified or decided by consensus between two independent raters to ensure objectivity.

4. **Report transparently**
   Clearly document the thresholding strategy in the methods section. For transparency and reproducibility, we recommend including a supplementary table listing the threshold value used for each participant, especially in cases where an individualized threshold was applied.

This structured approach enables consistency across the majority of participants while allowing flexibility for challenging cases. It also promotes transparency and interpretability in reporting DLMO estimation procedures.

References

Blume C, Cajochen C, Schöllhorn I, Slawik HC and Spitschan M (2024) Effects of calibrated blue–yellow changes in light on the human circadian clock. *Nature Human Behaviour* 8(3): 590–605.

Heinrichs HS and Spitschan M (2025) Within-subjects ultra-short sleep-wake protocol for characterising circadian variations in retinal function. *PloS one* 20(5): e0300405.

Kennaway DJ (2023) The dim light melatonin onset across ages, methodologies, and sex and its relationship with morningness/eveningness. *Sleep* 46(5): zsad033.

Molina TA and Burgess HJ (2011) Calculating the dim light melatonin onset: the impact of threshold and sampling rate. *Chronobiology international* 28(8): 714–718.

Murray JM, Stone JE, Abbott SM, Bjorvatn B, Burgess HJ, Cajochen C, Dekker JJ, Duffy JF, Epstein LJ, Garbazza C et al. (2024) A protocol to determine circadian phase by at-home salivary dim light melatonin onset assessment. *Journal of Pineal Research* 76(5): e12994.

Voultsios A, Kennaway DJ and Dawson D (1997) Salivary melatonin as a circadian phase marker: validation and comparison to plasma melatonin. *Journal of biological rhythms* 12(5): 457–466.
